# Identification of novel gene-based risk score for prognosis in prostate cancer

**DOI:** 10.1038/s41598-025-03800-3

**Published:** 2025-07-01

**Authors:** Huangwei Huang, Xia Sun, Peixin Li, Haoxin Cai, Lejia Xu, Benkang Shi, Sifeng Qu

**Affiliations:** 1 Department of Urology, Cheeloo College of Medicine, Qilu Hospital, Shandong University, Jinan, Shandong China; 2 University of Health and Rehabilitation Sciences, Qingdao, Shandong China; 3 Department of Pharmacology, School of Basic Medical Sciences, Cheeloo College of Medicine, Shandong University, Jinan, Shandong China; 4 Shenzhen Research Institute of Shandong University, Shandong University, Shenzhen, Guangdong China; 5 Medical Integration and Practice Center, Cheeloo College of Medicine, Shandong University, Jinan, Shandong China; 6 Key Laboratory of Urinary Precision Diagnosis and Treatment, Universities of Shandong, Jinan, Shandong China

**Keywords:** Gene-based risk score, Prostate cancer, ***VGF***, Immune landscape, Cancer, Computational biology and bioinformatics, Biomarkers, Urology

## Abstract

Tumor carcinogenesis and progression result from multiple genetic alterations in tumor cells. However, reliable biomarkers for prostate cancer classification remain limited, often leading to either overtreatment or inadequate treatment. Additionally, effective biomarkers for selecting patients who may benefit from immunotherapy are still lacking. Using data from TCGA-PRAD, we established gene selection criteria to develop a gene-based risk score. We identified a novel gene risk panel comprising six genes (*SSTR1*, *CA14*, *HJURP*, *KRTAP5-1*, *VGF*, and *COMP*) for prostate cancer risk classification. Patients in the high-risk group were associated with poor prognosis. The gene panel exhibited significantly enhanced predictive accuracy for progression-free survival compared to conventional clinicopathological parameters, including T stage, N stage, primary Gleason score, and secondary Gleason score. High-risk patients exhibited a higher tumor mutation burden. Notably, immune activity of CD8 + T cells, NK cells, and the type II IFN response was significantly lower in the high-risk group, indicating a more immunosuppressive environment. Furthermore, a nomogram combining the gene-based risk score with T stage and histological grade was constructed. The expression of genes in the gene-based risk score was further validated using clinical samples, and *VGF* was found to play a significant role in prostate cancer progression. The nomogram could serve as a valuable biomarker for distinguishing between high-risk and low-risk of PFS prostate cancer patients and for selecting patients who might benefit from immunotherapy.

**Supplementary Information:**

The online version contains supplementary material available at 10.1038/s41598-025-03800-3.

## Introduction

Prostate cancer (PCa) is the most commonly diagnosed cancer among men, and its global incidence continues to rise annually^1^. Radical prostatectomy is widely used to treat PCa patients without distant metastases. However, the outcome of PCa patients received ajurvant therapies post-surgery are diverse, necessitating a careful balance between the risk of disease progression and therapy-related morbidity^2^. Despite treatment, biochemical recurrence (BCR) occurs in 20–53% of patients within ten years, with approximately 29% eventually progressing to detectable tumor recurrence^3–5^. Therefore, identifying biomarkers for progression-free survival (PFS) is crucial for selecting patients who would benefit most from adjuvant treatment.

Tumor carcinogenesis and progression are driven by multiple genetic variations and mutations in tumor cells^6^. Tumor neoantigens, resulting from accumulated somatic mutations, are clinically correlated with tumor mutation burden (TMB)^7^. Tumor tissues comprise not only neoplastic cells but also stromal cells, immune cells, and the extracellular matrix, all of which contribute to the tumor microenvironment (TME). The TME plays a critical role in carcinogenesis and progression through mechanisms such as immune escape^7^. Evading tumor immune surveillance is now recognized as a hallmark of tumor development, leading to the investigation of therapeutic targets such as programmed cell death 1 (PD-1), programmed cell death 1 ligand 1 (PD-L1), and cytotoxic T lymphocyte-associated antigen-4 (CTLA-4)^8^. Over the past decade, there has been a significant increase in immunotherapy trials for various solid tumors, yielding promising results in colorectal, lung, and bladder cancers^9–11^. However, immune checkpoint inhibitors have shown limited response rates and modest clinical efficacy in PCa patients, with only a subset of those with specific genomic alterations benefiting from this therapeutic approach, as PCa is not highly immunogenic^12,13^. Consequently, reliable biomarkers are essential for selecting PCa patients who may benefit from individualized immune checkpoint inhibitor therapy.

In the present study, genomic data were downloaded from The Cancer Genome Atlas (TCGA) database. We identified a novel gene-based risk score comprising six genes for PCa patient risk classification. Importantly, this gene-based risk score demonstrated superior prognostic efficacy compared to the current T stage, N stage, primary Gleason grade (priGleason), and secondary Gleason grade (secGleason). We investigated the gene mutation profile and gene enrichment across different risk groups. Additionally, we examined the differences in the tumor immune microenvironment between the two risk groups, suggesting that high-risk patients may potentially benefit from immunotherapy. We further validated gene expression using clinical samples and explored the potential biological function of *VGF* in PCa.

This study presents a gene-based risk score that not only predicts prognosis, aiding in the selection of patients requiring more active treatment, but also identifies patients who might benefit from immunotherapy.

## Methods

### Dataset source acquisition

The training group data were obtained from The Cancer Genome Atlas (TCGA) - prostate adenocarcinoma (PRAD) (https://portal.gdc.cancer.gov/), including RNA sequencing data (fragments per kilobase of transcript per million fragments sequenced (FPKM) value) of gene expression, copy number variation (CNV), somatic mutation, and clinical information. The clinical information included age, tumor stage, lymph node status, Gleason score, biochemical recurrent status and so on. For validation, data of GSE54460 were sourced from the Gene Expression Omnibus (GEO, https://www.ncbi.nlm.nih.gov/geo/) and MSKCC2010 were downloaded from the cBioPortal (http://www.cbioportal.org/). Immune infiltrate data for PRAD tissues were obtained from the Cistrome Project (http://www.cistrome.org/) using the Tumor IMmune Estimation Resource version 2.0 (TIMER2.0)^14^.

### Identification of differentially expressed genes (DEGs)

Differentially expressed genes (DEGs) between normal prostate samples and PCa samples were identified using the “Limma” package with screening criteria of |log2 FC| > 2 and False Discovery Rate (FDR) < 0.05^15^. DEGs between Gleason ≤ 7 and Gleason > 7 samples were also explored using the “Limma” package with screening criteria of |log2 FC| > 1 and FDR < 0.05. Heatmaps of DEGs were generated using the “pheatmap” package, and volcano plots were created with the “Limma” package. The “VennDiagram” package was employed to identify overlapping DEGs between the two groups.

### Biomarker selection and gene-based risk score construction

Univariable Cox regression analyses were performed to assess the prognostic value of DEGs. Hazard ratios (HRs) > 1 were considered risk genes, while HRs < 1 were classified as protective genes. The correlation between DEGs and PFS was analyzed using univariate Cox regression with the R package “survival,” with candidate biomarkers screened at p values < 0.05. Multivariate Cox regression was then used to build a gene-based risk score, estimating regression coefficients according to the “Akaike Information Criterion”. The risk score for each PCa patient was calculated using the following formula:

$$Risk{\text{ }}score{\text{ }} = \sum \left( {coefficient{\text{ }} \times {\text{ }}expression{\text{ }}of{\text{ }}signature{\text{ }}gene} \right)$$

The median risk score served as the cutoff value to divide patients into high-risk and low-risk groups. Kaplan-Meier (KM) curves were drawn using the “survival” and “survminer” packages in R. The area under the receiver operating characteristic curve (AUC) was calculated to evaluate the prognostic ability of the gene-based risk score using the “survivalROC” package in R. Additionally, univariate and multivariate Cox regression analyses were performed to identify the prognostic values of the signature and clinicopathological features.

### TMB value calculation and functional enrichment analysis

TMB, defined as mutation frequency, was calculated by dividing the total gene mutations (including base substitutions, insertions, or deletions) by the average length of human exons (38 Mb). The “maftools” package was used to provide a comprehensive presentation of mutational information in the high- and low-risk groups.

The significance criteria for determining DEGs between high and low risk groups was set as the FDR < 0.05, and |log2 FC|> 1. Gene Ontology (GO) and Kyoto Encyclopedia of Genes and Genomes (KEGG) pathway enrichment analyses were performed to investigate molecular functions using the “clusterProfiler,” “enrichplot,” “org.Hs.eg.db,” and “ggplot2” packages^16–19^.

### Estimation of immune signatures, TME cell infiltration level and tumor purity

The stromal score, immune score, ESTIMATE score, and tumor purity were calculated to clarify the infiltration levels of lymphocytes in each sample. The single-sample gene-set enrichment analysis (ssGSEA) algorithm was used to quantify the relative abundance of immune signatures in the TME. The enrichment levels of 29 immune signatures were quantified for each sample, with gene sets representing each immune signature detailed in Table S6^16,20^. The “sparcl” R package was used to divide samples into high and low immunity groups for further analysis. A deconvolution algorithm [TIMER2.0, http://timer.cistrome.org/] was employed to analyze the association between mutations in the six gene-based risk score genes and six immune cell types^21^.

### Construction and validation of a predictive nomogram

A nomogram was constructed based on the multivariable Cox regression model incorporating T stage, N stage, priGleason, secGleason, and risk score to predict 1-, 2-, and 3-year PFS. The nomogram provides a graphical representation that links individual patient factors to predict PFS probability in PCa patients. The performance of the prognostic models was evaluated using receiver operating characteristic (ROC) analyses. Calibration of the model was assessed by comparing observed survival with predicted survival through calibration curve plotting. The nomogram and calibration plots were generated using the “rms” R package.

### Tissue specimens

This study was approved by the Medical Ethics Committee of the Shandong University School of Clinical Medicine. Ten human PCa tissue samples were collected from Qilu Hospital of Shandong University, with from all participants and/or their legal guardians. Research involving human research participants must have been performed in accordance with the Declaration of Helsinki.

### Cell lines and SiRNAs

Human PCa cell lines LNCaP, C4-2 and PC3 cells were purchased from Cell Resource Center, Institute of Biochemistry and Cell Biology at the Chinese Academy of Sciences, Shanghai, PR China. These cells were cultured in RPMI-1640 medium supplemented with 10% fetal bovine serum (FBS) and maintained at 37 °C with 5% CO2.

The small interfering RNAs (siRNAs) targeting *VGF* and control siRNA were obtained from Genepharma (Shanghai, China). The sequences for *VGF* siRNA1, *VGF* siRNA2, and negative control siRNA are listed in Table S7. Lipomaster 2000 Transfection Reagent (Vazyme, China) was used to transfect the siRNA into PCa cells. The experiment was performed according to the manufacturer’s instructions.

### RNA extraction, reverse transcribed and qPCR

Total RNA was isolated using SPARKeasy Cell RNA Kit (Sparkjade, China) and reverse transcribed using the SPARKscript II One Step RT-PCR Kit (Sparkjade, China). Real-time quantitative polymerase chain reaction (qPCR) was conducted using MagicSYBR Mixture (CWBIO, China) to measure the RNA levels of interest. Results were normalized to Beta Actin expression, and mRNA levels were calculated using the 2–ΔΔCt method. The primers used in this study are listed in Table S7.

### Western blot

Total proteins from PCa cells were extracted with RIPA lysis buffer containing proteinase inhibitor. The protein concentrations were measured by the BCA reagent kit (Beyotime, China). Proteins were separated by SDS-PAGE, transferred to polyvinylidene difluoride membranes (Merck Millipore, Germany), blocked in 5% nonfat milk, and incubated with primary antibodies against VGF (1:1000; Abways; O15240) and Beta Actin (1:7000; Proteintech; 66009-1-ig) at 4 °C overnight. Then, the membranes were washed in Tris-buffered saline with Tween, incubated with anti-mouse or anti-rabbit horseradish peroxidase-conjugated secondary antibody for 1 h at room temperature, and developed with the enhanced chemiluminescence method (Vazyme, China). Beta Actin served as a loading control.

### Cell proliferation assay

Cell proliferation was assessed with EdU, CCK-8 and Colony formation assays. The EdU assay was performed according to the protocol of the BeyoClick™ EdU Cell Proliferation Kit (Beyotime, China). Briefly, the treated cells were seeded in 96-well plates and incubated with 50 µM EdU for 2 h at 37 °C. After fixation with 4% paraformaldehyde, cells were exposed to 100 µL of 1× Apollo^®^ reaction cocktail and stained with 5 µg/mL Hoechst 33,342 for nuclear visualization. Images were captured using a fluorescence microscope (Olympus, Tokyo, Japan), and the percentage of EdU-positive cells was defined as the proliferation rate. For the CCK-8 assay, the treated cells were seeded in 96-well plates and incubated with 100 µL 10% CCK-8 solution for 3 h at 37 °C. Absorbance was measured at 450 nm and 630 nm using a FlexA-200 spectrophotometer (Allsheng, Hangzhou, China). In the colony formation assay, 800 cells were seeded in 6-well plates, and cell colonies (> 50 cells/colony) were counted following 1% crystal violet staining after 10 days. All experiments were performed in triplicate and repeated three times.

### Flow cytometry assay for the cell cycle

Cells were cultured and transfected with the corresponding siRNA for 48 h, then transferred to collection tubes. Cells were fixed in 1 mL of 70% cold ethanol overnight, washed three times with ice-cold PBS, and suspended in 100 µL PBS with 0.5 mL PI/RNase Staining Buffer (BD Company) at room temperature in the dark for 30 min. Flow cytometry experiments were conducted within 1 h after PI staining. Cell cycle distribution was analyzed using a flow cytometer (Beckman Coulter, Brea, CA, USA).

### Statistical analysis

All analyses were conducted using the R v.4.2.0 and SPSS v.25 software. KM method was used to estimate the influence of the hub genes on survival. Data from the two groups were compared using a two-tailed unpaired Student’s t-test. Categorical data were analyzed using the chi-square test. The correlation between continuous variables was assessed using the Spearman’s correlation analysis. All statistical tests were two-sided, and p-values < 0.05 or adjusted p-values < 0.05 were considered statistically significant (*p < 0.05, **p < 0.01, ***p < 0.001).

## Results

### Identification of prognostic genes in PCa development and progression

**Fig. 1:**
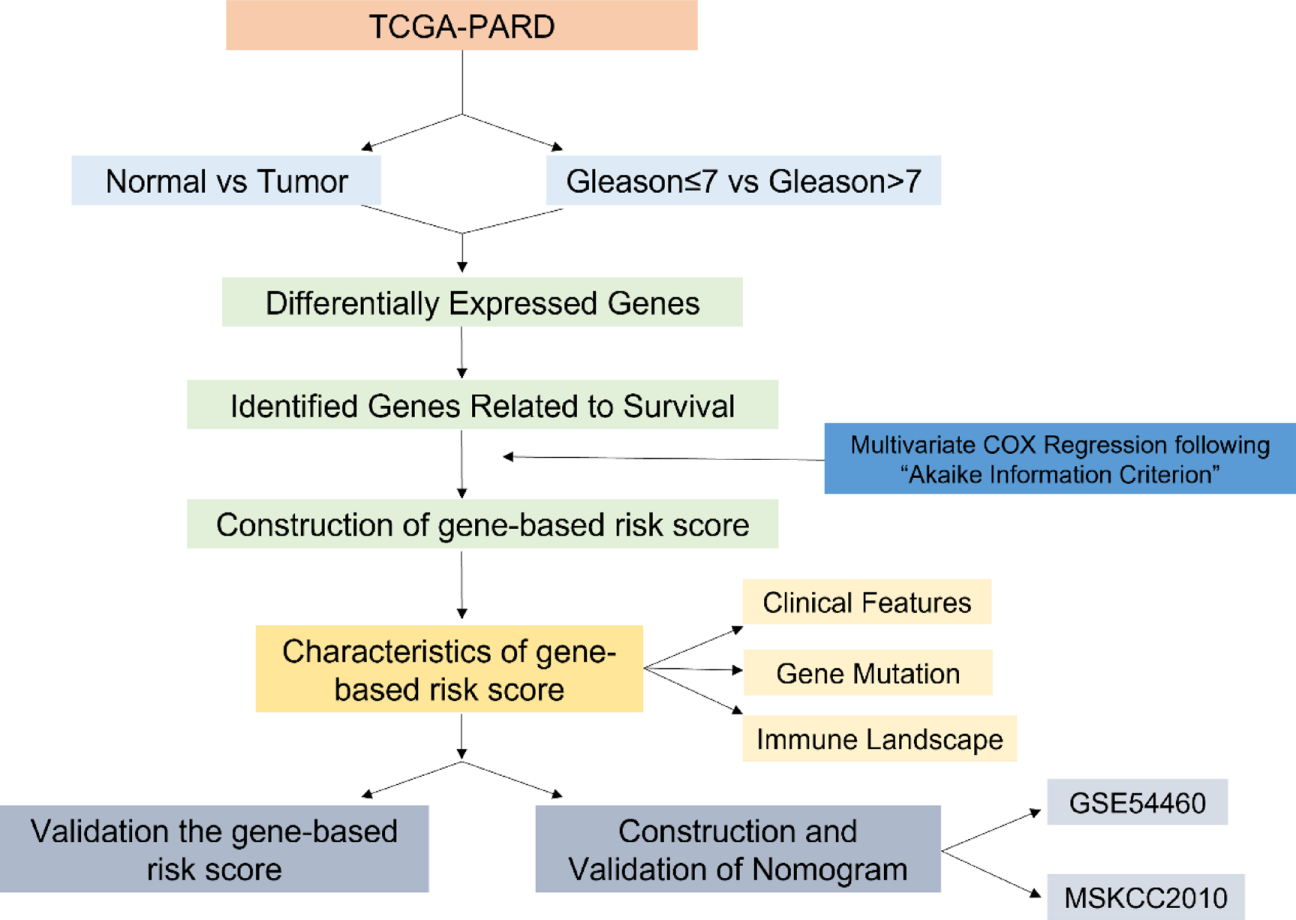
A workflow chart illustrating the process of establishing a gene-based risk score in prostate cancer.

**Fig. 2:**
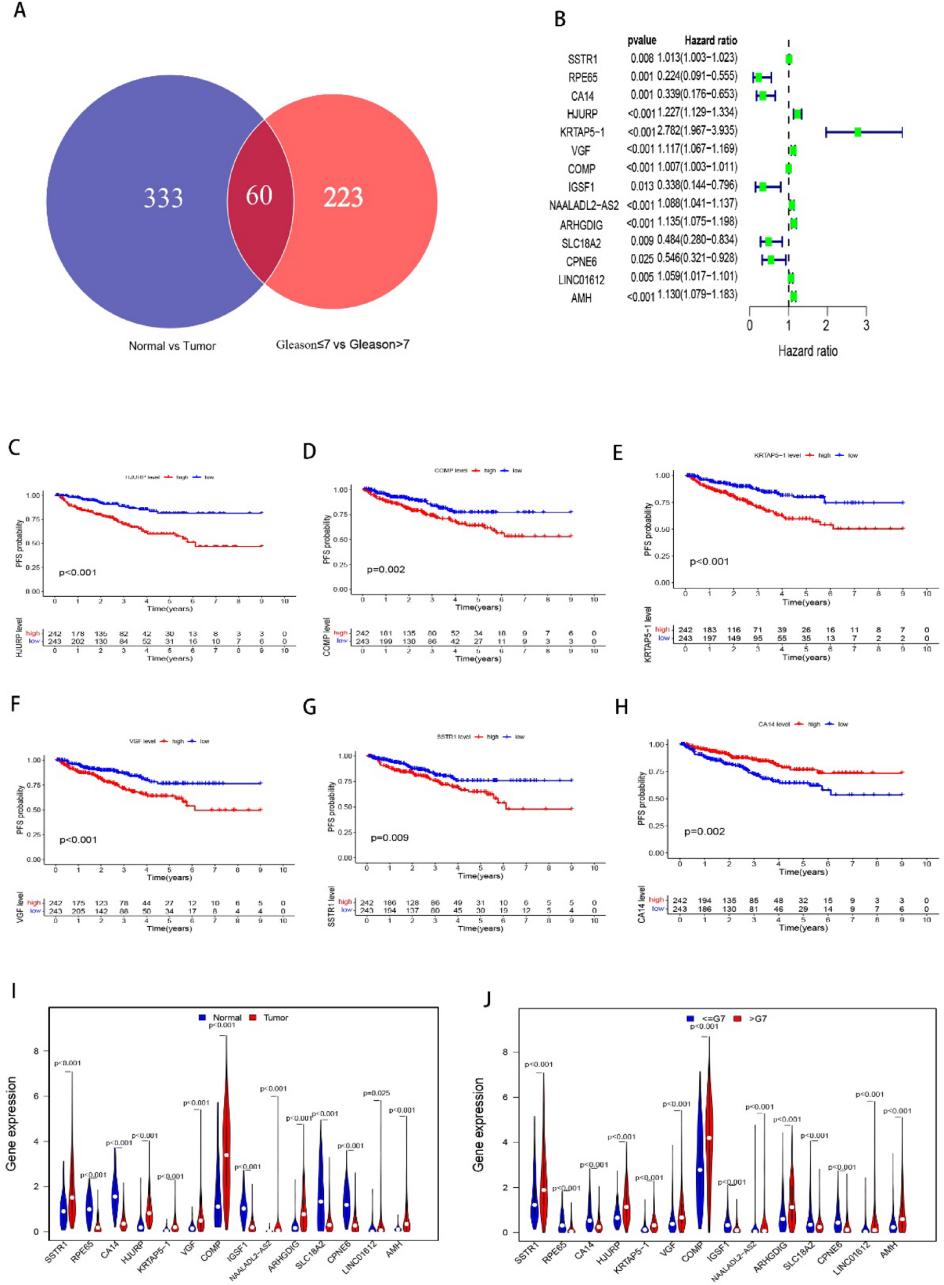
Identification of Prognostic Genes in PCa Development and Progression. (**A**) Venn diagram showing the overlap of DEGs between the different comparisons. (**B**) Univariable Cox regression analysis of DEGs correlated with PFS in PCa patients. (**C**)-(**H**) Kaplan-Meier survival analysis of PCa patients with high and low expression of DEGs: (**C**) HJURP, (**D**) COMP, (**E**) KRTAP5-1, (**F**) VGF, (**G**) SSTR1, and (**H**) CA14. (**I**) Comparison of DEG expression correlated with PFS between normal prostate samples and PCa samples. (**J**) Comparison of DEG expression correlated with PFS between Gleason ≤ 7 samples and Gleason > 7 samples.

Whole-exome sequencing data and clinical information for PCa patients were obtained from TCGA-PRAD (540 samples). DEG were generated using the “limma” package. To identify key genes involved in PCa carcinogenesis and progression, we compared gene expression between two cohorts: normal prostate samples versus PCa samples, and Gleason ≤ 7 samples versus Gleason > 7 samples (Fig. 1A). We identified 393 DEGs when comparing PCa samples to normal samples (**Figure S1A**, **Table S1**, |log2(FC)| >2 and FDR < 0.05, 51 normal samples and 489 tumor samples) and 283 DEGs when comparing Gleason ≤ 7 samples to Gleason > 7 samples (**Figure S1B**, **Table S2**, |log2(FC)| >1 and FDR < 0.05, 290 samples with Gleason score ≤ 7, 196 samples with Gleason score > 7). DEGs common to both comparisons could be functionally implicated in PCa initiation and progression. Using a Venn diagram, we filtered these genes, ultimately identifying 60 DEGs, including 39 up-regulated and 21 down-regulated genes, for further analysis **(**Fig. 2A, **Table S3)**.

We performed univariate Cox proportional hazards regression and Kaplan-Meier analyses on the 60 selected DEGs. Fourteen of these genes were found to be significantly correlated with the PFS of PCa patients **(**Fig. 2B, **Table S4)**. Overexpression of nine genes, including *HJURP*, *COMP*, *KRTAP5-1*, *VGF*, *SSTR1*, *LINC01612*, *NAALADL2-AS2*, *AMH*, and *ARHGDIG*, was significantly associated with poorer prognosis in PCa patients **(p < 0.05**, Fig. 2C-G, S1 C-1F**)**. Conversely, overexpression of *CA14*, *RPE65*, *IGSF1*, *SLC18 A2* and *CPNE6* was significantly correlated with better prognosis **(p < 0.05**, Fig. 2H, S1G-J).

Moreover, mRNA expressions of *CA14*, *RPE65*, *IGSF1*, *SLC18 A2* and *CPNE6* were significantly lower in PCa samples compared to benign samples, while *HJURP*, *COMP*, *KRTAP5-1*, *VGF*, *SSTR1*, *LINC01612*, *NAALADL2-AS2*, *AMH* and *ARHGDIG* were elevated in tumor samples **(p < 0.05**, Fig. 2I**)**. When patients were divided into two groups based on Gleason score (Gleason ≤ 7 and Gleason > 7), similar gene expression patterns were observed **(**Fig. 2J**)**. These findings suggest that these 14 genes may play crucial roles in PCa carcinogenesis and progression, making them promising candidates for predicting PCa prognosis.

### Establishment and evaluation of gene-based risk score

**Fig. 3:**
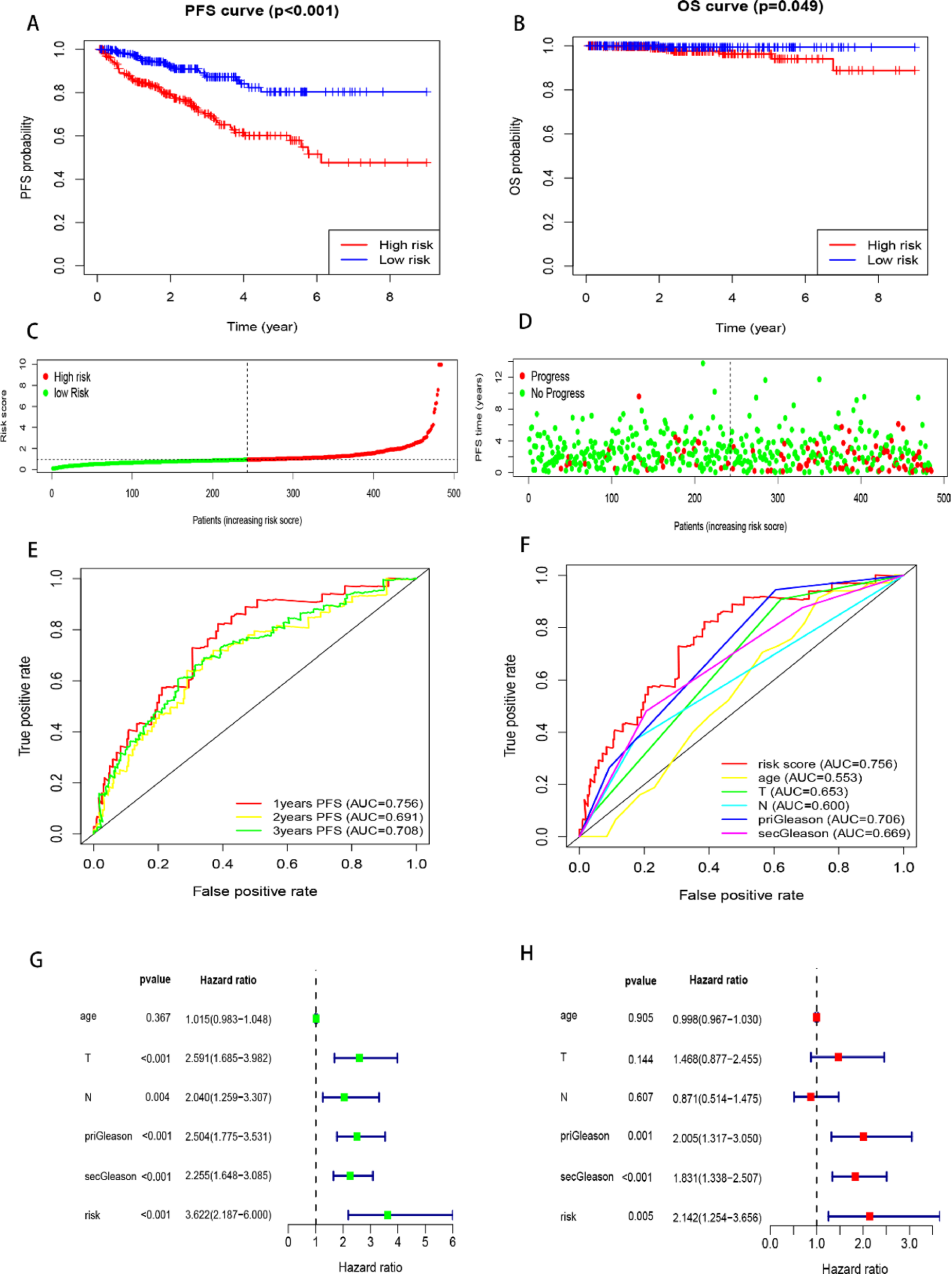
Characterization of the Gene-based Risk Score. (**A**) Kaplan–Meier survival curve of PFS based on the gene-based risk score in the TCGA database. (**B)** Kaplan–Meier survival curve of OS based on the gene-based risk score in the TCGA database. (**C**-**D**) Evaluation of the relationship of the gene-based risk score with PFS. (**E**) ROC curves of the gene-based risk score for predicting PFS at 1, 2, and 3 years. (**F**) ROC curves comparing the predictive performance of the gene-based risk score and other clinical characteristics for PFS at 1 year. (**G**-**H**) Univariate and multivariate Cox regression analyses of the gene-based risk score and clinical indexes.

Multivariate Cox regression analysis was used to identify the most suitable genes as potential predictive biomarkers, leading to the establishment of a gene-based risk score based on the “Akaike Information Criterion”. The final panel comprised six genes (*SSTR1*, *CA14*, *HJURP*, *KRTAP5-1*, *VGF* and *COMP*), with their coefficients listed in Table S5. To evaluate the prognostic value of this gene panel, PCa patients from the TCGA cohort were divided into low-risk and high-risk groups. Kaplan-Meier curves demonstrated that patients in the high-risk group had poorer outcomes compared to those in the low-risk group, both in terms of PFS and overall survival **(**Fig. 3A and B**)**. The risk curve further illustrated significant differences between the two groups **(**Fig. 3C-D**)**.

The AUC for PFS at 1-, 2-, and 3-year intervals was 0.756, 0.691, and 0.708, respectively, as determined by ROC analysis **(**Fig. 3E**)**. Furthermore, the AUC comparison for different PFS criteria at 1-, 2-, and 3-year intervals revealed that the gene-based risk score provided superior predictive efficacy compared to current clinical characteristics (e.g., T stage, N stage, priGleason, secGleason) **(**Fig. 3F, S2 A-B**)**. Univariate and multivariate Cox analyses confirmed that the gene-based risk score is an independent prognostic factor with greater efficacy than other clinical characteristics **(**Fig. 3G-H**)**.

### Characteristics of clinical features of PCa patients with gene-based risk score

**Fig. 4:**
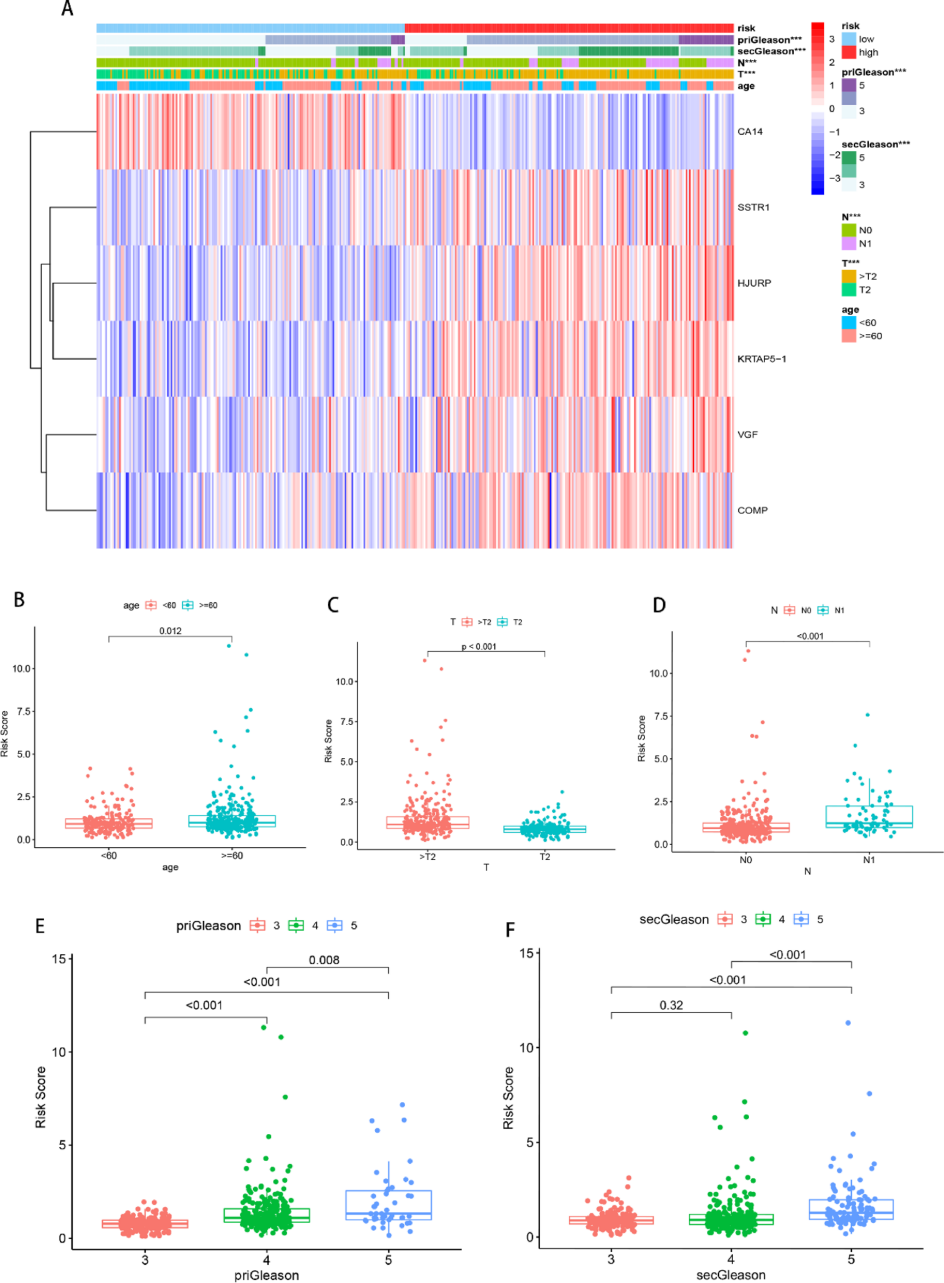
Characteristics of Clinical Features in PCa Patients with the Gene-based Risk Score. (**A**) Heatmap showing the expression levels of the six genes in low-risk and high-risk PCa patients. Patient annotations include priGleason, secGleason, N status, T status, and age. (**B**-**F**) Association of the risk score with clinical features: (**B**) older age, (**C**) advanced tumor stage, (**D**) positive regional lymph node status, (**E**) higher priGleason, and (**F**) higher secGleason.

Comparing high-risk and low-risk groups revealed distinct differences in tumor T stage, N stage, priGleason, and secGleason, with higher expression of gene in the gene-based risk score observed in high-risk patients, except for CA14, which exhibited an opposite expression pattern **(**Fig. 4A**)**. Further analysis of risk scores in various clinical subgroups showed that older patients (≥ 60 years) had higher risk scores than younger patients **(**Fig. 4B**)**. Additionally, higher risk scores were associated with higher T stage and N stage, indicating more aggressive and metastatic disease **(**Figs. 4C-D**)**. According to the analysis of patients with different Gleason scores (either primary or secondary), it suggested that patients with higher risk score would more frequently to be diagnosed with higher Gleason score **(**Fig. 4E, F**)**. Moreover, higher risk scores were correlated with higher total Gleason scores (totalGleason) **(Figures S3 A)**. Principal components analysis (PCA) demonstrated that benign and malignant prostate samples could be distinctly separated using the risk gene panel **(Figures S3B)**, and a similar distinction was observed among PCa samples with different Gleason scores **(Figures S3 C)**. These results indicate that the risk score increases during PCa progression, with the high-risk group associated with more malignant behavior.

### Gene mutation profiles and DEGs in different risk groups

**Fig. 5:**
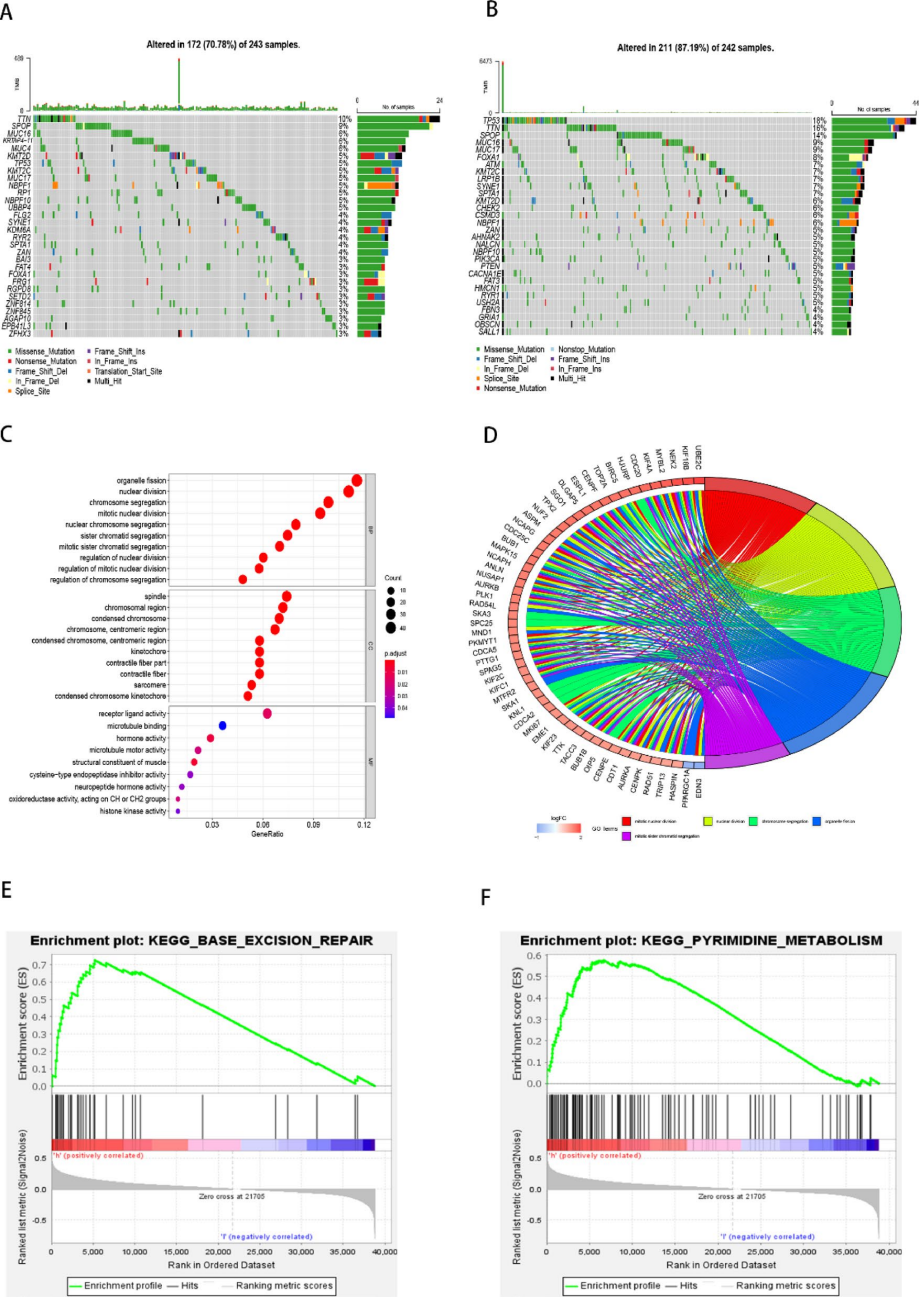
Gene Mutation Profiles and DEGs in Different Risk Groups. (**A**) Waterfall plot showing the top 30 mutated genes in the low-risk group. (**B**) Waterfall plot showing the top 30 mutated genes in the high-risk group. (**C**-**D**) Gene Ontology (GO) functional analysis of differentially expressed genes between high-risk and low-risk groups, categorized by: BP (biological process), CC (cellular component), and MF (molecular function). (**E**-**F**) KEGG pathway analysis of DEGs between high-risk and low-risk groups, highlighting the top-ranked pathways: (**E**) top 1 and (**F**) top 2 pathways.

Whole-exome sequencing data from TCGA-PRAD were analyzed using the “maftools” package to comprehensively present mutation information. The top 30 most frequently mutated genes in both high-risk and low-risk groups are depicted in the waterfall plot, with clear differences between the two groups (Fig. 5A-B). Some genes were common to both groups but with varying mutation rates. For example, the mutation rate of TP53 was higher in the high-risk group (18%, Fig. 5B) compared to the low-risk group (5%, Fig. 5A). Additionally, certain mutations, such as those in ATM, LRP1B, CHEK2, AHNAK2, PI3 KCA, and PTEN, were unique to the high-risk group.

To further investigate DEGs between the two risk groups, the “limma” package was used **(Figure S4 A-B**, |log2(FC)| >1 and FDR < 0.05**)**. GO enrichment analysis was conducted to explore the biological functions of these DEGs. Significant enrichment was observed in biological processes such as organelle fission, nuclear division, mitotic nuclear division, and sister chromatid segregation **(**Fig. 5C-D, S4 C**).** GSEA-KEGG analysis of high- and low-risk groups, based on the TCGA-PRAD dataset, revealed that the high-risk group was significantly associated with pathways such as base excision repair, pyrimidine metabolism, cell cycle, homologous recombination, and DNA replication (FDR < 0.05, Fig. 5E-F, S4D-F). Cancer sample in high-risk group upregulate base excision repair pathway activity to mitigate oxidative DNA damage, thereby enhancing their survival under genotoxic stress conditions (Fig. 5E). Pyrimidine metabolism, a critical branch of nucleotide biosynthesis, supplies precursors for DNA/RNA synthesis and fuels proliferative demand in cancer cells. Dysregulation of this pathway—a hallmark of malignancy—enrichment in high-risk group (Fig. 5F). These findings suggest that the functions of DEGs between the two risk groups are closely linked to the cell cycle and may play a significant role in gene mutation.

### Characteristics of immune landscape with gene-based risk score

**Fig. 6:**
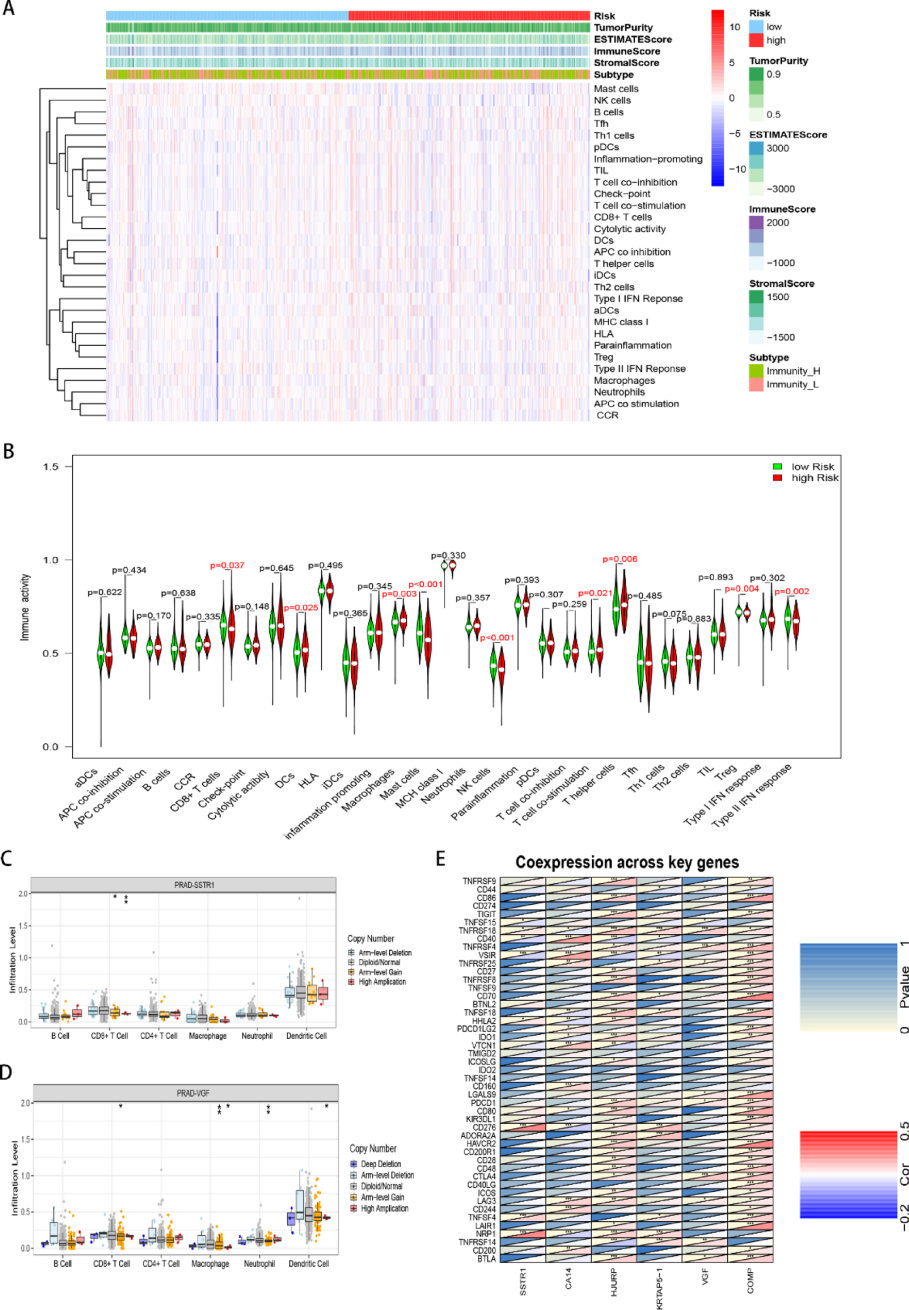
Characteristics of the Immune Landscape with the Gene-based Risk Score. **(A**) Heatmap showing the enrichment of 29 immune signatures in the two risk score groups. Patient annotations include immunity cluster, tumor purity, ESTIMATE score, immune score, and stromal score. **(B**) Differential activity of the 29 immune signatures between the two risk score groups. **(C**-**D)** Association of mutations in (**C**) SSTR1 and (**D**) VGF with six immune cell types. Data was obtained from the TIMER website (https://cistrome.shinyapps.io/timer/). **(E**) Correlation between genes in the gene-based risk score and immune checkpoints. Red in the bottom right corner indicates positive correlation, blue indicates negative correlation, and yellow in the top left corner indicates statistical significance. *p < 0.05, **p < 0.01, ***p < 0.001.

To explore the potential relationship between immunity and the gene-based risk score, stromal score, immune score, ESTIMATE score, and tumor purity were analyzed. No significant differences were observed between the two risk groups in stromal scores, immune scores, ESTIMATE scores, or tumor purity **(Figure S5 A-D)**. Further investigation using ssGSEA scores allowed us to divide samples into two clusters: immunity low and immunity high. The immune cell types, functions, pathways, and differences in 29 immune-associated gene sets were then identified between these two clusters (**Table S6**,** Figure S5E-F**). We next examined the immune landscape characteristics across different risk score groups **(**Fig. 6A**)**. Although no significant difference was found in the overall immune landscape enrichment between the two risk groups **(Figure S5G)**, specific immune subtypes revealed notable differences. The immune activity of regulatory T cells (Treg) was significantly lower in the high-risk group, while the immune activity of dendritic cells (DCs), macrophages, T cell co-stimulation, and T helper cells was significantly higher **(**Fig. 6B**)**. But It was interesting found that the immune activity of CD8 + T cells, NK cells and response of type II IFN was significantly lower in high-risk group **(**Fig. 6B**)**. Additionally, we explored the immune characteristics of each of the six genes in the gene-based risk score using the TIMER-PRAD database. *SSTR1* and *VGF* amplification were negatively associated with CD8 + cells (Fig. 6C-D).

We also examined the co-expression of the six genes in the gene-based risk score with several immune checkpoints. The results indicate a correlation between the gene-based risk score genes and immune checkpoints, such as CTLA-4. The immune checkpoint molecule CTLA-4 demonstrated significant positive correlations with *HJURP*, *VGF* and *COMP* expression **(p < 0.05;** Fig. 6E), suggesting potential coordinated regulation of immune evasion and tumor progression pathways. Notably, the expression of certain immune checkpoints, including CTLA-4, was elevated in the high-risk group **(Figure S5H)**. Collectively, these findings suggest that although the immune score varies among PCa patients, reflecting the heterogeneity of PCa, patients in the high-risk group are more prone to maintaining a suppressive TME, leading to poor prognosis.

### Validation the gene-based risk score and construction of a nomogram

**Fig. 7:**
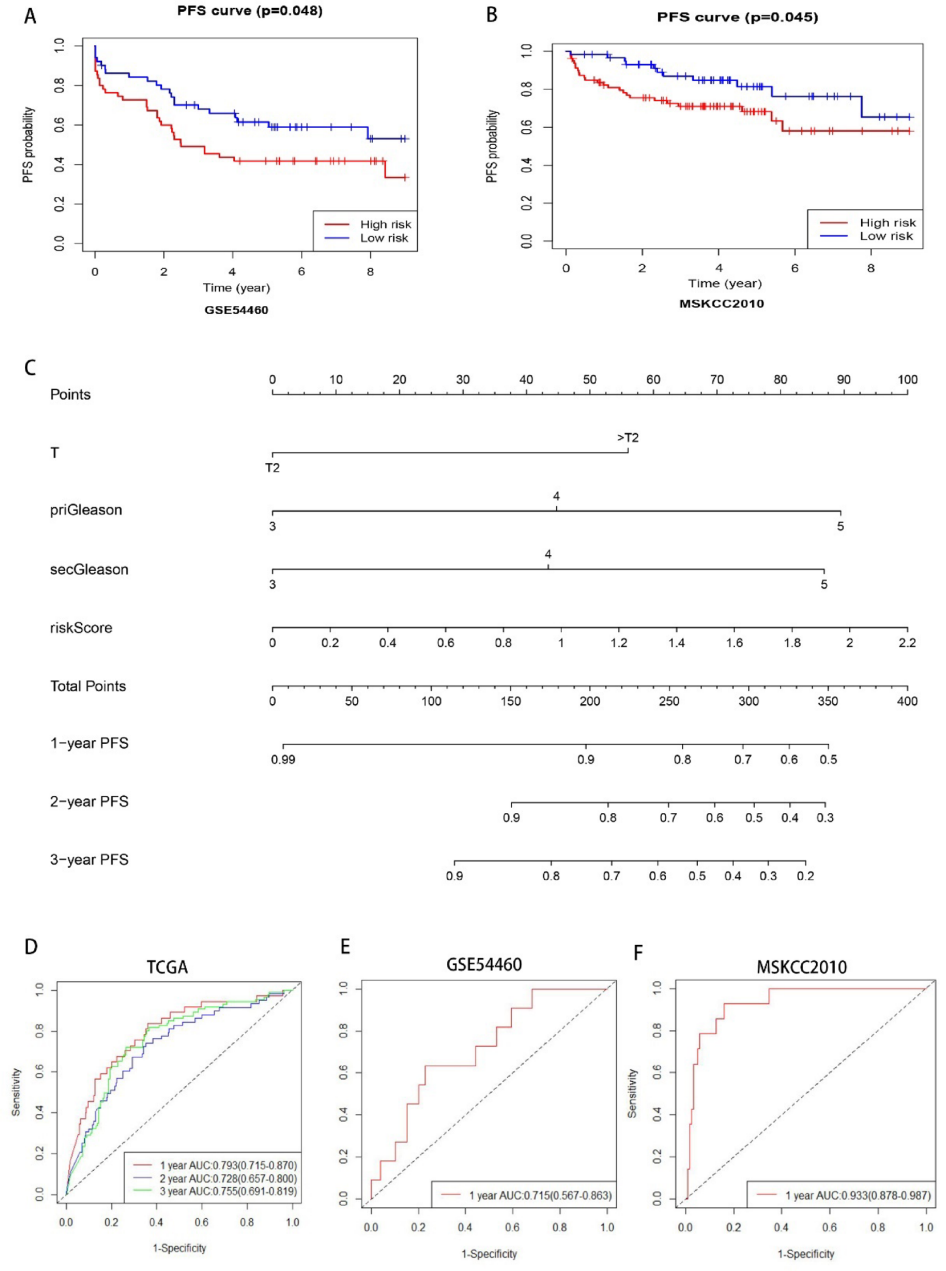
Validation of the Gene-based Risk Score and Construction of a Nomogram. (**A**-**B**) Kaplan-Meier curves of PFS for PCa patients in high- and low-risk score groups within the (**A**) GSE54460 cohort and (**B**) MSKCC2010 cohort. (**C**) The constructed nomogram for predicting the PFS probabilities of PCa patients. (**D**) ROC curves of the nomogram for predicting 1-,2- and 3-year PFS of PCa patients within TCGA cohort. (**E**) ROC curves of the nomogram for predicting 1- year PFS of PCa patients within GSE54460 cohort. (**F**) ROC curves of the nomogram for predicting 1- year PFS of PCa patients within MSKCC2010 cohort.

The predictive value of the gene-based risk score was validated using two independent datasets, GSE54460 and MSKCC2010 **(GSE54460 cohort: 106 samples**,** MSKCC cohort: 198 samples**, Fig. 7A-B**)**. The results demonstrated that PCa patients with a high risk score had significantly worse PFS than those with a low risk score. Furthermore, the nomogram was constructed by combining the gene-based risk score with T stage, and and histological grade (including priGleason and secGleason) **(**Fig. 7C**)**. The AUCs of the nomogram for predicting the 1-, 2- and 3-year PFS were 0.793 (95% CI: 0.715–0.870), 0.728 (95% CI: 0.657–0.800) and 0.755(0.691–0.819), respectively, in TCGA cohort **(**Fig. 7D**)**. The calibration plot suggested that the predicted 1-, 2-, and 3-year PFS rates were consistent with the actual PFS rates within an acceptable margin of error in PCa **(Figure S5I)**. The predictive performance of the nomogram was externally validated in two independent cohorts (GSE54460 and MSKCC2010). The nomogram demonstrated discriminative ability, with AUCs for predicting 1-year PFS of 0.715 (95% CI: 0.567–0.863) in the GSE54460 cohort and 0.933 (95% CI: 0.878–0.987) in the MSKCC2010 cohort (Fig. 7E-F). The nomogram represents a practical clinical instrument for predicting PFS in PCa patients, with nomogram scores correlating with prognosis and informing therapeutic decision-making.

### The role of *VGF* in PCa progression

**Fig. 8:**
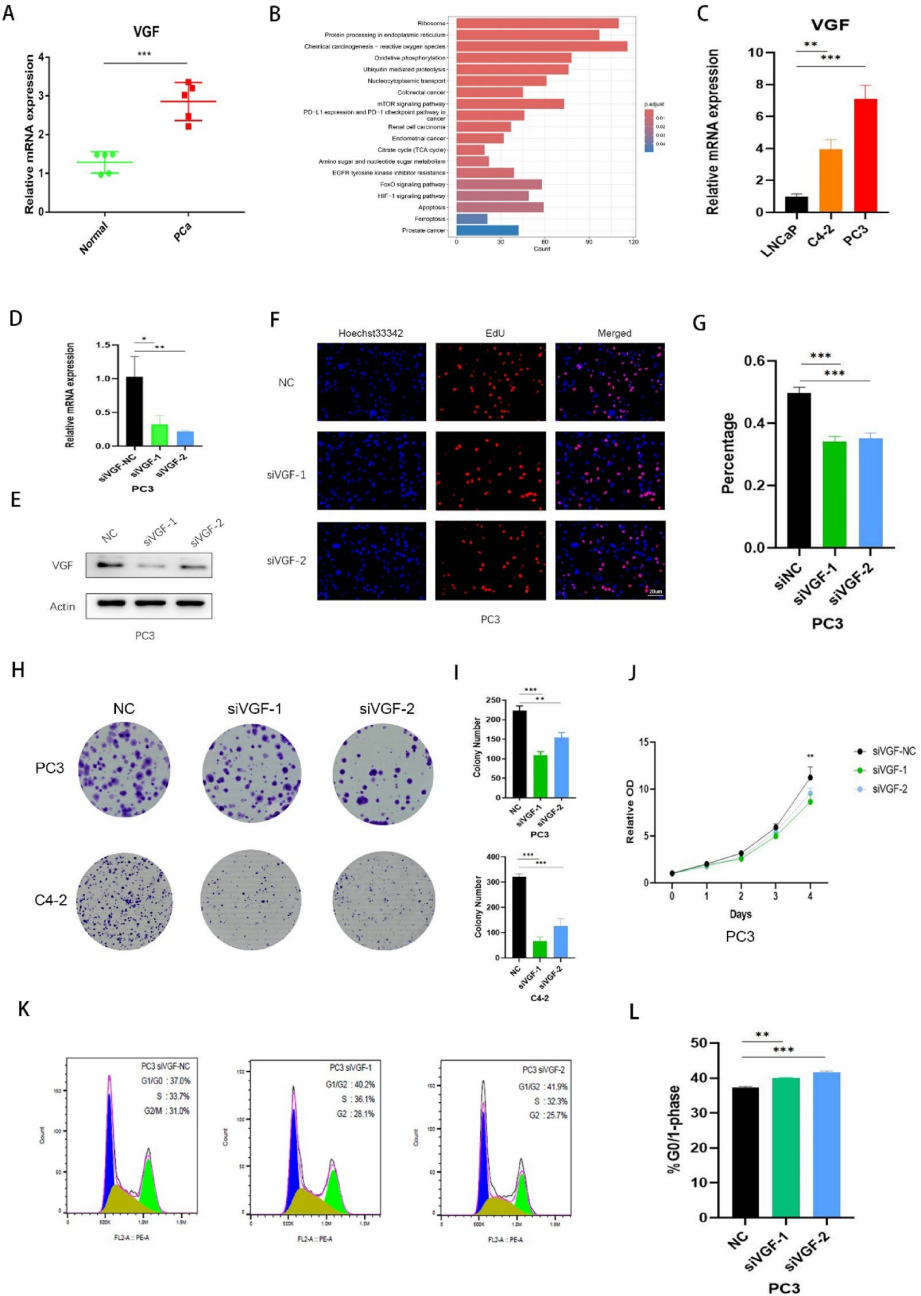
The Role of VGF in Prostate Cancer Progression. (**A**.) Expression levels of VGF in normal prostate samples compared to PCa samples. (**B**) KEGG pathway enrichment analysis of DEGs comparing high- and low-VGF expression groups in the TCGA-PRAD cohort, (**C**) Expression levels of VGF in PCa cell lines. (**D)** Quantification of VGF levels in PC3 cells using qPCR, comparing negative control (NC) with siVGF-1/2 treatments. (**E**) Western blot analysis of VGF protein expression in PC3 cells transfected with siNC or siVGF. The original blots are presented in Supplementary Fig. 7. (**F**-**G**) EdU assay analyzing cell proliferation in PC3 cells transfected with VGF siRNA or NC. Scale bar: 20 μm. (**H**-**I**) Colony formation assay showing the colony-forming ability of treated PCa cells, with representative results displayed. **(J**) CCK-8 assay assessing the proliferation ability of PC3 cells. **(K**-**L**) Flow cytometric analysis of cell cycle arrest in PC3 cells treated with siVGF and NC. All data are presented as means ± SD. **p < 0.01; ***p < 0.001; ****p < 0.0001.

The expression of the genes in the gene-based risk score was validated using clinical samples. *VGF*, *HJURP*, *SSTR1* and *COMP* were found to be expressed at higher levels in PCa tissues compared to normal prostate tissues. Conversely, *CA14* expression was higher in normal prostate tissues. While *KRTAP5-1* mRNA expression did not show a statistically significant difference between normal and PCa samples (*p* = 0.074), there was a trend toward higher expression in PCa tissues (Fig. 8A, S6 A-E). *VGF*, initially characterized in neurons and neuroendocrine cells, plays critical roles in normal metabolic processes, cell survival, and hippocampal proliferation^22,23^. However, emerging evidence has shifted focus toward its tumor-promoting functions in cancer biology^24^. Despite these advances, the specific contribution of *VGF* to prostate cancer (PCa) progression remains unclear. KEGG pathway enrichment analysis of DEGs comparing high- and low-*VGF* expression groups in the TCGA-PRAD cohort demonstrated significant involvement of key oncogenic pathways, particularly the mTOR signaling pathway and the PD-L1 expression/PD-1 checkpoint pathway in cancer (Fig. 8B).

Using multiple PCa cell lines, we found that *VGF* expression was higher in PC3 and C4-2 cells compared to LNCaP cells (Fig. 8C). To further validate the role of *VGF* in PCa progression, VGF was depleted in PC3 and C4-2 cells using specific siRNAs. The efficiency of *VGF* knockdown was confirmed by qPCR and Western blotting (Fig. 8D-E, S6 F). EdU assays showed a significant reduction in cell proliferation in both PC3 and C4-2 cells following *VGF* knockdown (Fig. 8F-G, S6G-H). Colony formation and CCK-8 proliferation assays further supported the role of *VGF* in promoting tumor proliferation (Fig. 8H-J, S6I). Moreover, *VGF* knockdown resulted in a marked arrest of cells in the G0/G1 phase of the cell cycle compared to control PC3 cells (Fig. 8K-L). These findings suggest that *VGF* plays a significant role in PCa progression, particularly in promoting cell proliferation, and that it could serve as a potential therapeutic target in PCa.

## Discussion

Despite recent therapeutic advancements, PCa remains a major urological malignancy associated with significant morbidity and mortality^25,26^. The aggressiveness of PCa varies widely, ranging from localized indolent disease to rapidly progressive, lethal, and metastatic forms^27–29^. Therefore, identifying patients who require either active surveillance or timely intervention is crucial, highlighting the urgent need for reliable biomarkers. In this study, we identified a gene panel that may play a critical role in PCa carcinogenesis and progression. The risk score based on this gene panel effectively classified PCa patients into high-risk and low-risk groups with distinct prognoses.

Most of the genes in gene-based risk score was found to be involved in malignant development and progression. For example, *HJURP*, a molecular chaperone of centromere protein-A, promotes chromosome separation and mitosis. Wenjie Lai et al. reported that *HJURP* can increase *CDKN1 A* degradation via the *GSK3β/JNK* signaling pathway to enhance PCa cell proliferation^30^. Additionally, *HJURP* is correlated with poor outcomes in ovarian and breast cancers^31,32^. *COMP*, another gene in the panel, is a prognostic factor and biomarker in colon cancer, where it promotes cell proliferation by activating the *AKT* pathway^33,34^. *COMP* has also been shown to initiate cancer stem cells through the activation of *Jagged1-Notch3* signaling^35^. *SSTR1* is implicated in colon cancer and is associated with aggressive disease features^36^. *VGF*, highly expressed in a subgroup of lung adenocarcinomas, has been linked to *EGFR-TKI* resistance and epithelial-to-mesenchymal transition^24^. *CA14* may act as a tumor suppressor in PCa, but its specific functions and mechanisms remain unclear^37^. When used in combination, these six genes efficiently distinguished high-risk from low-risk PCa patients. The gene-based risk score demonstrated superior prognostic value compared to current clinical parameters such as T stage, N stage, and Gleason score (priGleason and secGleason). Furthermore, both the total gene mutation rate and single gene mutation rates (e.g., *TP53*, *FOXA1*, and *ATM*) were significantly higher in the high-risk group, consistent with reports that higher gene mutation rates are associated with more aggressive PCa, particularly in key cancer-associated genes^38^. Biological function analyses further supported the observation that the high-risk group is more involved in cell proliferation, including processes such as the cell cycle and DNA repair, indicating a more aggressive disease type.

In recent years, immunotherapies have shown great promise in treating various cancers, including skin, bladder, lung, and kidney cancers, as well as tumors deficient in mismatch repair, providing extremely durable responses for some patients^39–49^. However, numerous clinical trials utilizing CTLA-4 or PD-1/PD-L1 inhibitors in patients with metastatic castration-resistant PCa (mCRPC) have been disappointing, with limited survival benefits when administered as monotherapy in unselected patients^12,13,45,50,51^. In addition to PD-L1 expression, high TMB has been proposed as a predictive biomarker for response to immune checkpoint inhibitors^52–54^. Our gene-based risk score revealed that the high-risk group had a higher TMB than the low-risk group. The most frequently mutated gene in the high-risk group was *TP53*, which has been demonstrated to be a driver mutation that directly affects tumor growth^55^. Co-occurring mutations in *TP53* and other genes (e.g., *EGFR*, *STK11*, or *KRAS*) have also been shown to have predictive value for immune checkpoint inhibitors^56–58^. Moreover, Qiang Zhang et al. demonstrated that inhibition of *ATM* sensitizes pancreatic cancer to immune checkpoint blockade therapy^59^. The high frequency of single-gene mutations and the enrichment of DNA synthesis and repair pathways in the high-risk group suggest that these patients may have a higher TMB, potentially making them more responsive to immune checkpoint inhibitors.

To further explore the TME in different risk groups, we conducted an analysis of TME status. The TME, a complex and dynamic multicellular environment, plays a critical role in tumor development. Interactions between tumor cells and the surrounding stroma significantly influence tumor initiation, progression, and metastasis^60,61^. Tumor-infiltrated immune cells are highly associated with tumorigenesis, angiogenesis, and metastasis. Due to the immunosuppressive microenvironment and the heterogeneous nature of PCa, identifying molecular subtypes and investigating TME characteristics are crucial for predicting responses to immunotherapy. Our ssGSEA analysis showed that the immune activity of CD8 + T cells, NK cells, and the type II IFN response was lower in the high-risk group of the gene-based risk score. CD8 + T cells and NK cells are key players in the immune response against cancer cells, with CD8 + T cells differentiating into cytotoxic T lymphocytes that eliminate cancer cells, and NK cells playing a crucial role in immune surveillance, with their infiltration in tumor tissues being prognostic in some patient cohorts^62,63^. Schreiber and colleagues have shown that low immunogenicity and failure of immune detection lead to aggressive carcinogenesis in multiple organs, with the IFN-γ signaling pathway playing a critical role in governing antitumor immune responses^64,65^. The reduced immune activity of CD8 + T cells, NK cells, and the type II IFN response in the high-risk group impairs the ability to eliminate tumors, leading to disease progression. Furthermore, we investigated the co-expression of the six genes in the gene-based risk score with immune checkpoints. The results indicate that the expression of key genes is correlated with immune checkpoints, such as CTLA-4. Collectively, these findings suggest that patients in the high-risk group have an immunosuppressive TME, and restoring anticancer immunity could enable these patients to benefit from immunotherapy.

Validation of gene expression using clinical samples further demonstrated that gene expression levels are consistent with TCGA data. *VGF*, originally identified in neurons and neuroendocrine cells, is responsible for normal metabolism as well as cell survival and proliferation in the hippocampus^22,23^. Previous studies on *VGF* have primarily focused on nervous system diseases, with only a few studies linking *VGF* to lung cancer^24^, pancreatic cancer^66,67^, and breast cancer^68^. However, the roles of *VGF* and the other five genes in PCa are not well understood. Our study has demonstrated that *VGF* plays an important role in PCa proliferation. It is plausible that the other genes may have similar functions, as suggested by bioinformatic data, though this remains a limitation of the current study.

At present, the CARPA score is the most commonly used prediction model for prostate cancer recurrence, which mainly considers clinical and pathological information(PSA, Gleason score, T-stage and percent positive biopsies)^69^. Classification analysis based on a large number of samples that can better reflect tumor features and heterogeneity becomes possible with the advent of high-throughput sequencing. Therefore, it is important to pay attention to the genomic information of tumor samples while considering the clinical and pathological information. The c-indices for CAPRA were 0.66 (0.57–0.75)^69^, and the C-indices of the nomogram in this study were 0.739 (0.682–0.796). The limitation of this model is that the initial PSA and the percent positive biopsies were not recorded in the TCGA database, so the clinical information of these two factors was not taken into account in the nomogram. Several limitations need to be acknowledged. The conclusions were generated based on bioinformatic data, and further validation of the expression of the six genes should be conducted with larger patient samples. Additionally, the biological functions of these genes and their relationship with immune cells should be further investigated.

## Conclusions

In summary, we successfully developed a gene-based risk score comprising six genes, which can effectively classify PCa patients into high-risk and low-risk groups. Notably, high-risk patients demonstrated significantly reduced PFS, elevated TMB, and an immunosuppressive TME. Among the six genes, targeting VGF showed promising potential as a therapeutic approach. Therefore, the nomogram combining the gene-based risk score with T stage and histological grade not only distinguishes more aggressive PCa patients but also serves as a valuable tool for guiding patient treatment and selecting biomarkers for immunotherapy.

## Electronic supplementary material

Below is the link to the electronic supplementary material.

Supplementary Material 1

## Data Availability

The datasets used during this study can be downloaded from public databases including TCGA (https://portal.gdc.cancer.gov/), GEO (https://www.ncbi.nlm.nih.gov/geo/) and cBioPortal (http://www.cbioportal.org/).

## Abbreviations

PCa: Prostate cancer
BCR: Biochemical recurrence
PFS: Progress-free survival
TMB: Tumor mutation burden
TME: Tumor microenvironment
PD-1: Programmed cell death 1
PD-L1: Programmed cell death 1 ligand 1
CTLA-4: Cytotoxic T lymphocyte-associated antigen-4
TCGA: The Cancer Genome Atlas
priGleason: primary Gleason grade
secGleason: Secondary Gleason grade
PRAD: Prostate adenocarcinoma
FPKM: Fragments per kilobase of transcript per million fragments sequenced
CNV: Copy number variation
TIMER2.0: Tumor IMmune Estimation Resource version 2.0
DEGs: Differentially expressed genes
FDR: False discovery rate
HRs: Hazard ratios
KM: Kaplan-Meier
AUC: Area under receiver operating characteristic curve
GO: Gene Ontology
KEGG: Kyoto Encyclopedia of Genes and Genomes
ssGSEA: Single-sample gene-set enrichment analysis
ROC: Receiver operating characteristic
siRNAs: Small interfering RNAs
qPCR: Real-time Quantitative polymerase chain reaction
totalGleason: Total Gleason score
PCA: Principal components analysis
Treg: Regulatory T cells
DCs: Dendritic cells
NC: Negative control
