# Supplementary material for: Identification of novel gene-based risk score for prognosis in prostate cancer
Source: Sci Rep. 2025 Jul 1;15:22128. doi: 10.1038/s41598-025-03800-3 (PMC12219829; doi:10.1038/s41598-025-03800-3)
Supplement: Supplementary file 1 — Supplementary Material 1 [file 41598_2025_3800_MOESM1_ESM.pdf]

Supplementary Material

Identification of novel gene-based risk score for prognosis in prostate cancer

Huangwei Huang[1,2,3], Xia Sun[4], Peixin Li[1],Haoxin Cai[4], Lejia Xu[4], Benkang Shi[1,2*], Sifeng Qu[5,6*]

[1]Department of Urology, Qilu Hospital, Cheeloo College of Medicine, Shandong University, Jinan, Shandong, China
[2]Key Laboratory of Urinary Precision Diagnosis and Treatment in Universities of Shandong, Jinan, Shandong, China
[3]University of Health and Rehabilitation Sciences, Qingdao, Shandong, China
[4]Department of Pharmacology, School of Basic Medical Sciences, Cheeloo College of Medicine, Shandong University, Jinan, Shandong, China
[5]Shenzhen Research Institute of Shandong University, Shandong University, Shenzhen, Guangdong, China
[6]Medical Integration and Practice Center, Cheeloo College of Medicine, Shandong University, Jinan, Shandong, China

* Correspondence:
Benkang Shi
bkang@sdu.edu.cn
Xia Sun
sunxia@sdu.edu.cn

Supplementary Figures：

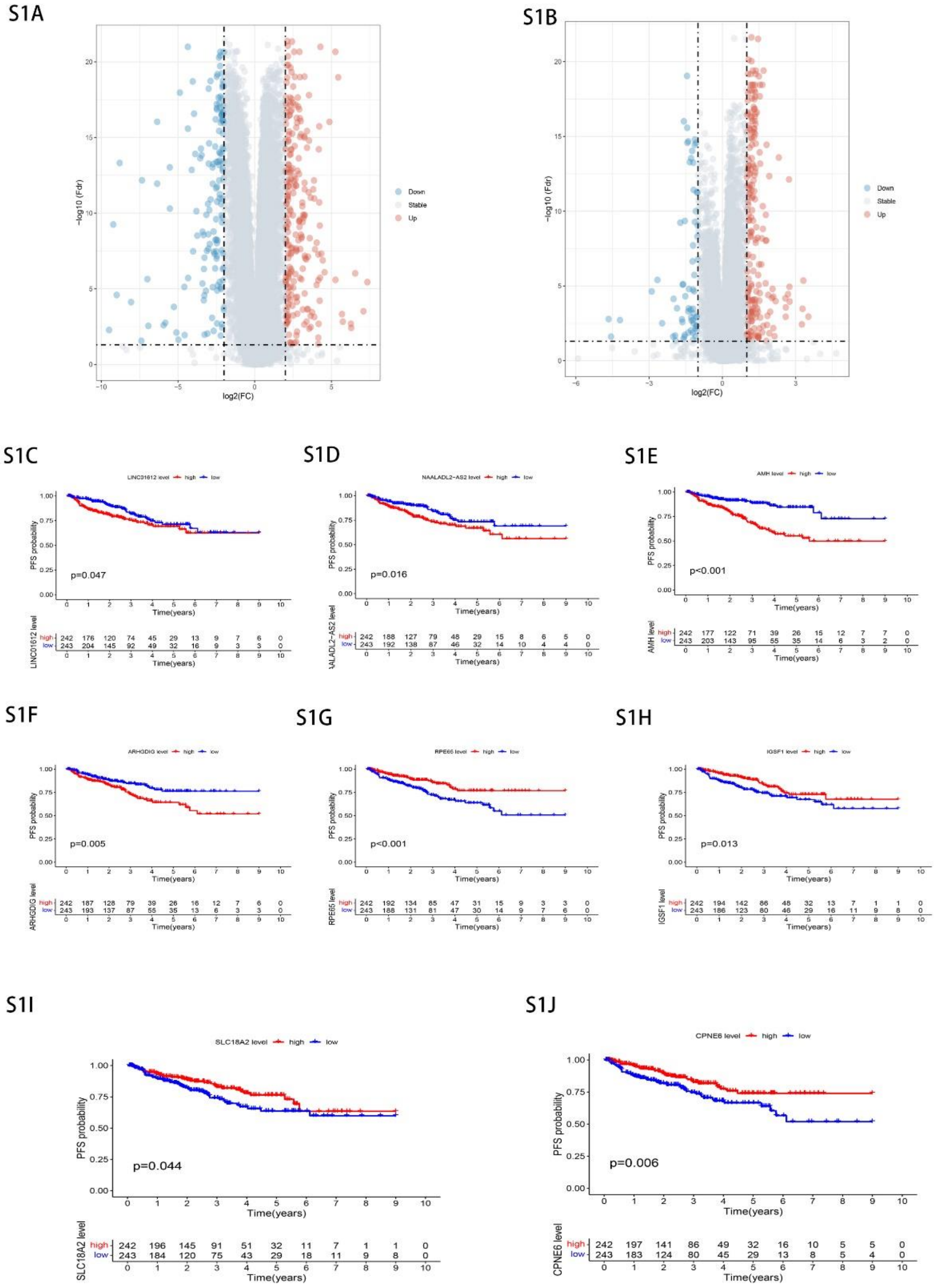

Supplementary Figure 1. (S1A) Volcano plot of differentially expressed genes (DEGs) between the normal prostate samples and PCa samples of TCGA-PRAD database (|log2(FC)| >2 and FDR<0.05); (S1B) Volcano plot of DEGs between the Gleason≤7 samples and Gleason>7 samples from TCGA-PRAD database (|log2(FC)| >1 and FDR<0.05);(S1C-S1J) Survival analysis of PCa patients with high and low expression

of DEGs. Kaplan-Meier curves of (S1C) LINC01612, (S1D) NAALADL2-AS2, (S1E) AMH, (S1F) ARHGDIG, (S1G) RPE65, (S1H) IGSF1,(S1I) SLC18A2 and (S1J) CPNE6.

S2A

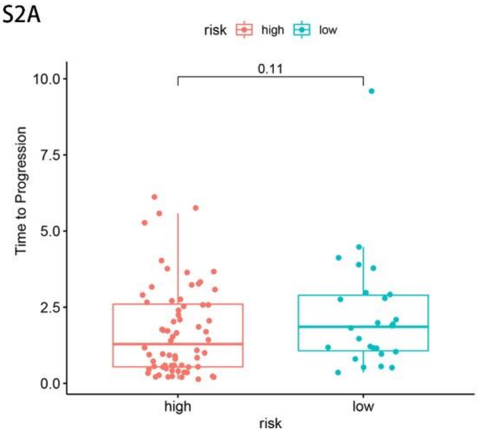

S2B

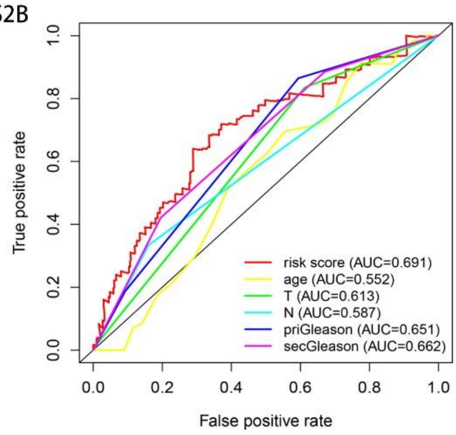

S2C

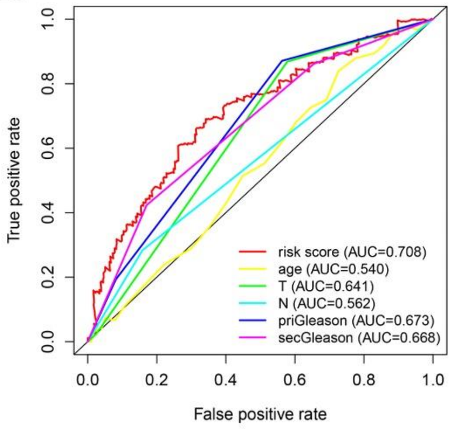

Supplementary Figure 2. (S2A) Evaluation of the relationship of the risk score patterns with time to progression; (S2B) The ROC curves of risk score pattern and other clinical characteristics for PFS at 3 years; (S2C) The ROC curves of risk score pattern and other clinical characteristics for PFS at 3 years.

S3A

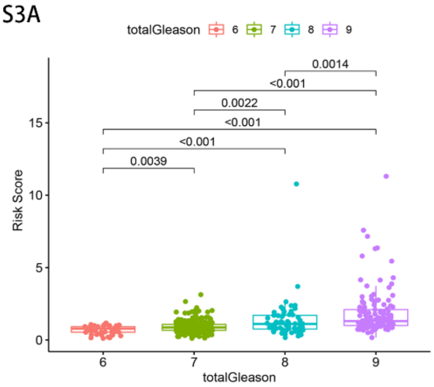

S3B

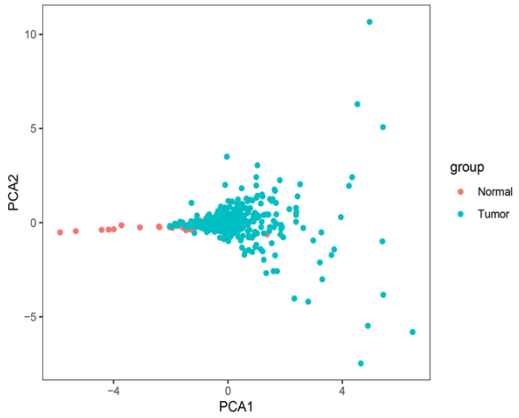

S3C

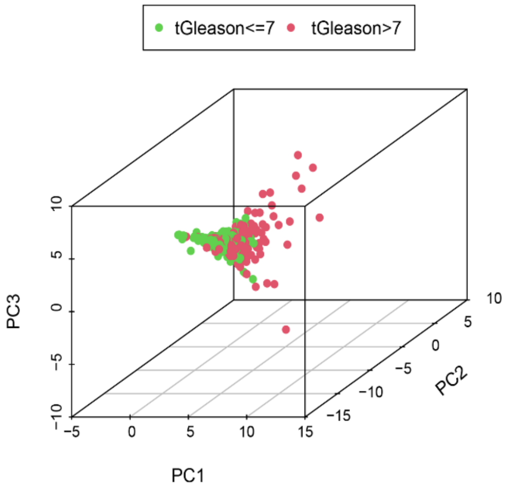

Supplementary Figure 3. (S3A) Risk score was associated with total Gleason grade.; (S3B) PCA analysis show that prostate benign and malignant samples could be separated from each other using the risk gene panel; (S3C) PCA analysis show that total Gleason score <=7 and >7 samples could be separated from each other using the risk gene panel.

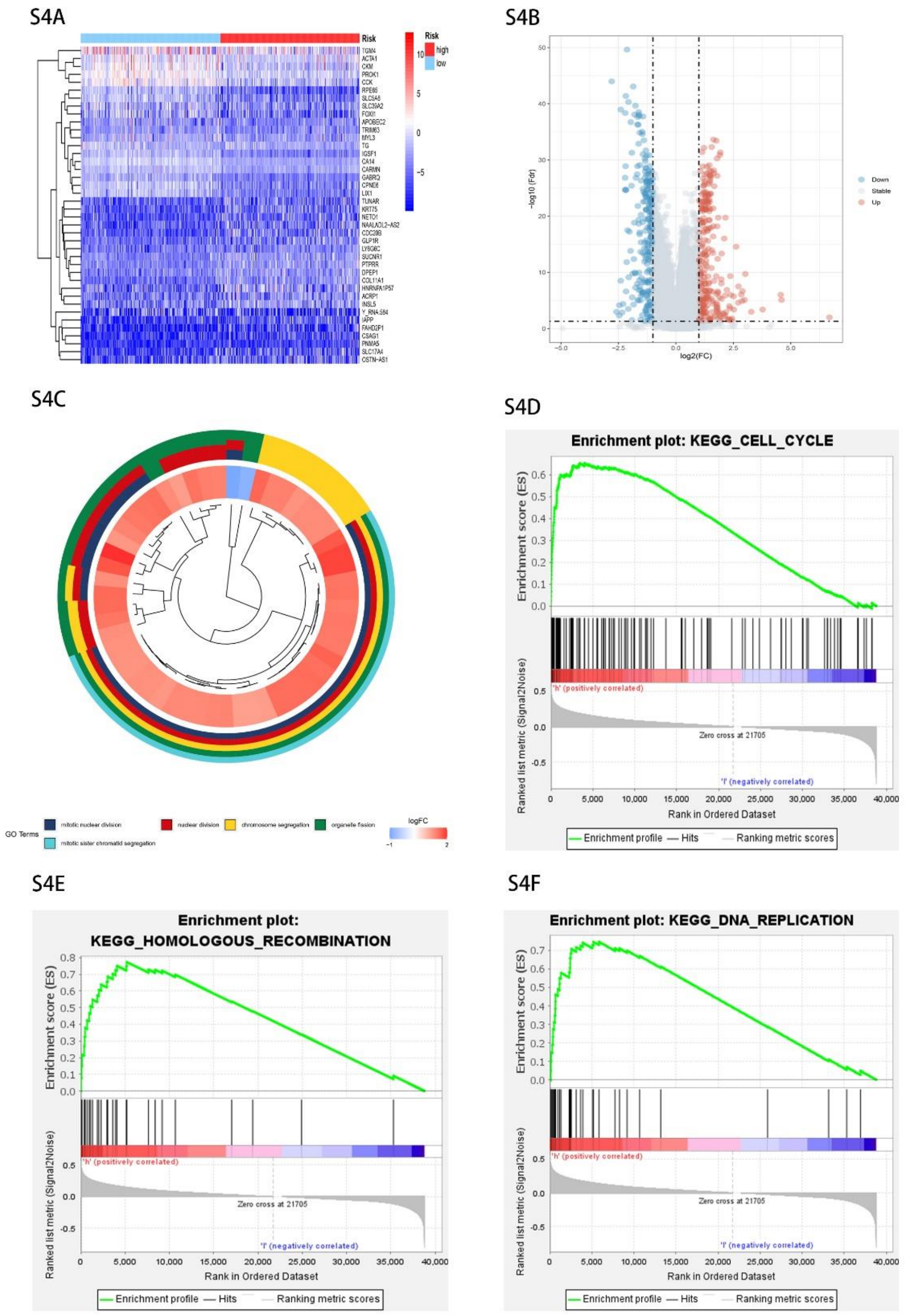

Supplementary Figure 4. (S4A-S4B) The differentially expressed gene between the

two risk groups; (S4C) GO enrichment analysis of differentially expressed genes between the two risk groups; (S4D-S4F) KEGG pathway of differentially expressed genes between high- and low-risk group.

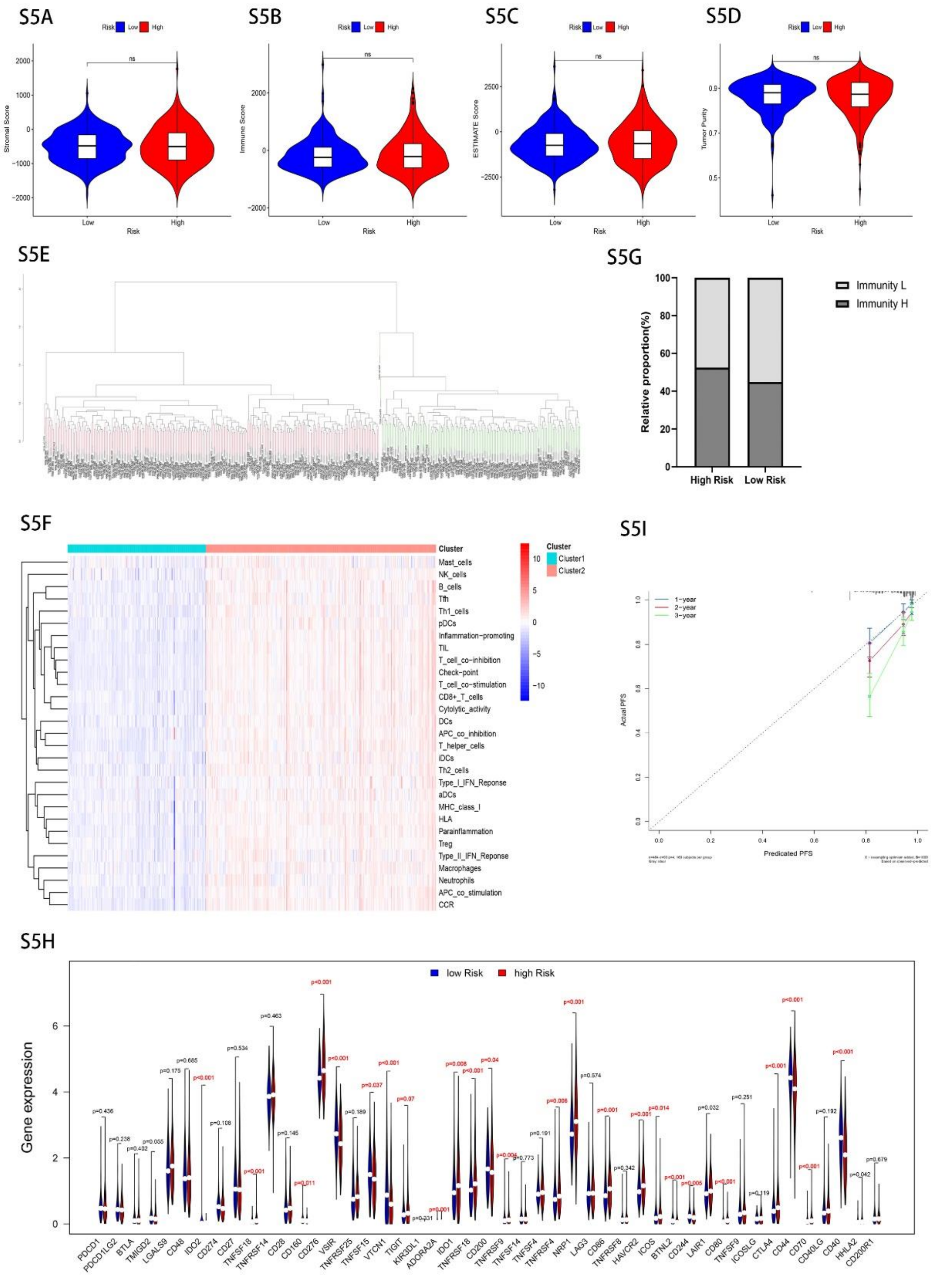

Supplementary Figure 5. (S5A-S5D) The analysis of the stromal score, immune score,

ESTIMATE score, and tumor purity between the high- and low-risk groups; (S5E) ssGSEA score divided samples into two clusters, immunity low and high; (S5F) The differences in 29 immune-associated gene sets were shown in two distinct immunity clusters; (S5G-S5H) The immune landscape in two risk groups; (S5I) Calibration blots indicated the agreement between the predicted and actual PFS at 1-, 2-, and 3-years intervals.

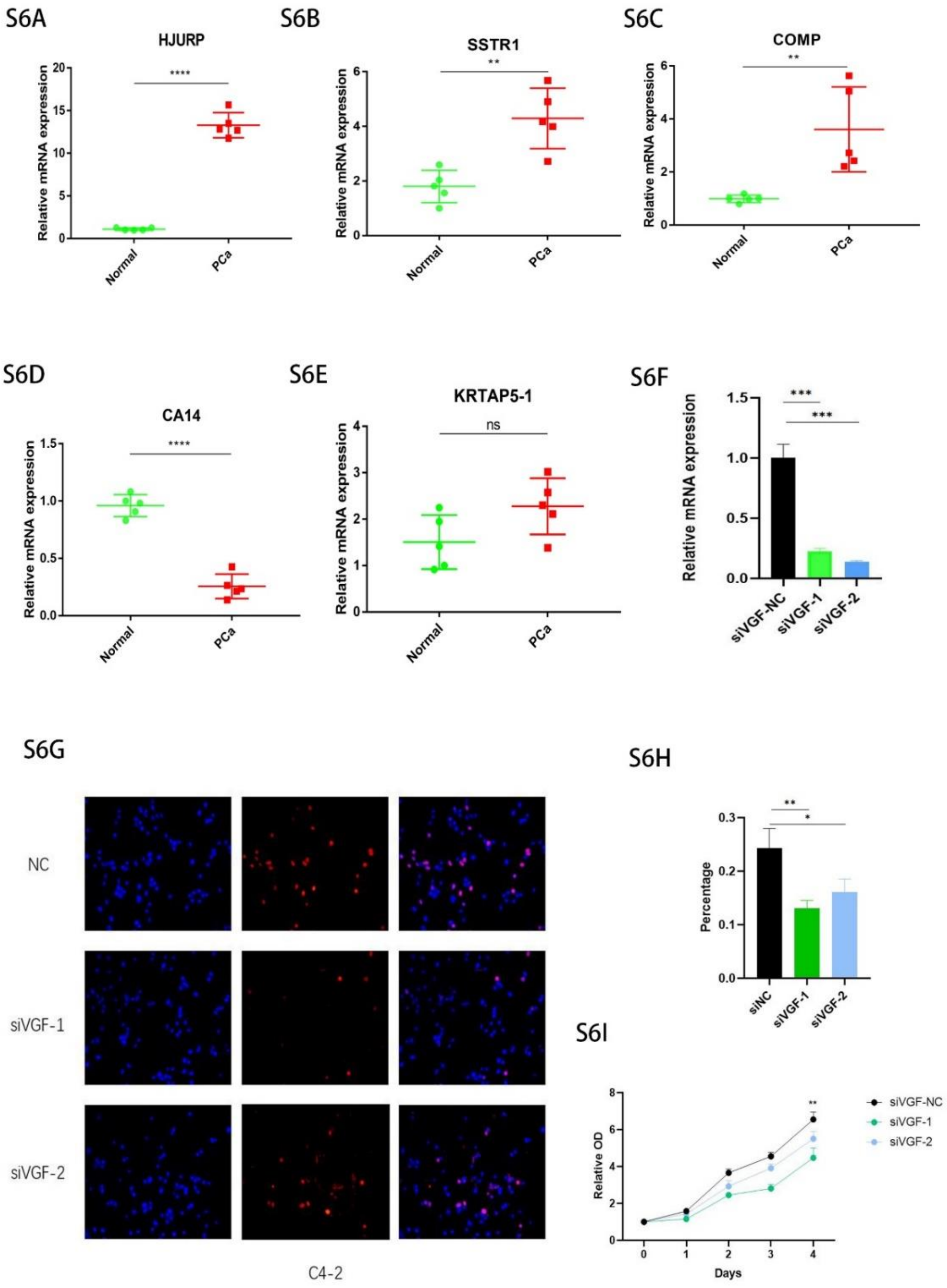

Supplementary Figure 6. (S6A-S6E) The expression of risk score pattern genes

(*HJURP*, *SSTR1*, *COMP*, *CA14*, and *KRTAP5-1*) in normal prostate samples and prostate cancer samples; (S6F) The levels of VGF in C4-2 cells were measured by qRT-PCR, negative control (NC) vs siVGF-1/2; (S6G-S6H) EdU analysis of the cell proliferation ability in C4-2 cells transfected with siRNA of VGF or the NC; (S6I) CCK-8 assay was used to detect C4-2 cell proliferation ability. All data are presented as means ± SD. **p < 0.01; ***p < 0.001; ****p < 0.0001.

Supplementary Figure 7

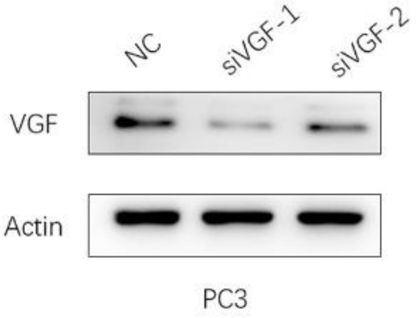

VGF

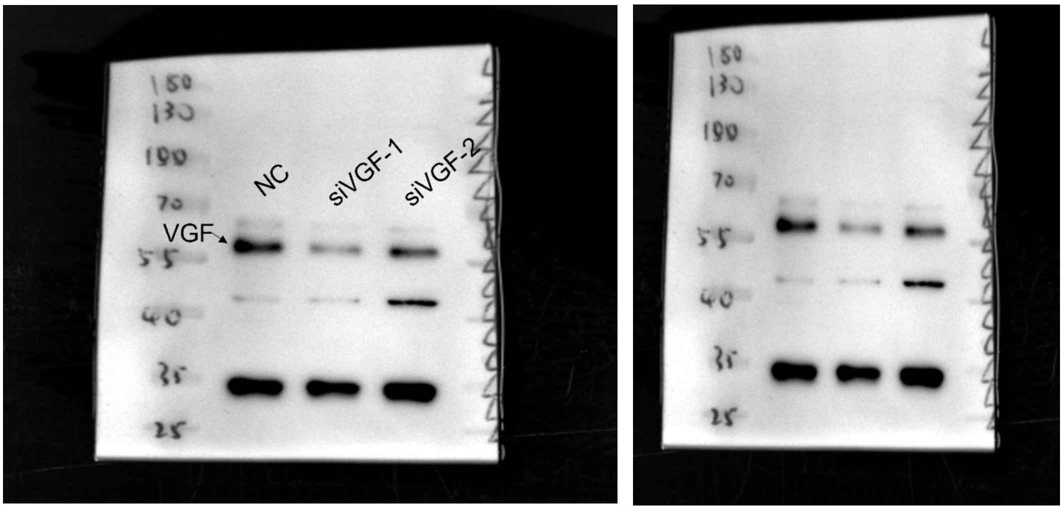

ACTIN

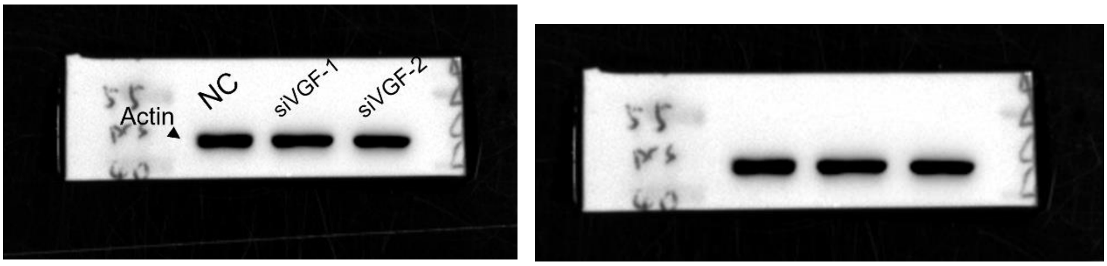

Supplementary Figure 7 Original blots of Figure 8D.The 3 bands are siVGF-NC, siVGF-1, siVGF-2 in sequence for PC3 cell.

Supplementary Tabel 1. 393 DEGs when comparing PCa samples to normal samples

gene	Normal Sample Mean	Tumor Sample Mean	logFC	pValue
SPC25	0.531734	1.162491	1.128443	3.58E-15
PPP1R1A	1.079668	0.535007	-1.01296	3.22E-11
ODF3L2	0.165456	0.333184	1.009872	1.06E-05
INSL5	0.234476	1.398308	2.576172	1.27E-05
CDC25C	0.278669	0.649581	1.220955	2.58E-18
CDC20	1.799942	4.530641	1.331765	1.67E-19
MUCL1	0.506915	4.8361	3.254029	0.001335
ITLN1	0.328921	0.147271	-1.15927	3.66E-06
BUB1	0.453121	1.125699	1.312853	1.17E-21
TTK	0.66106	1.368288	1.049519	5.69E-13
FRG2HP	1.679397	0.780353	-1.10575	0.011227
PRAME	0.242906	0.659258	1.440441	0.011302
MCM10	0.144134	0.36719	1.349116	3.07E-15
CENPA	0.408133	1.186134	1.539155	4.74E-22
RUNDC3A	0.217651	0.505356	1.215286	1.85E-05
SYT4	0.601135	2.551671	2.085682	0.002585
SSTR1	3.054463	8.437271	1.465858	2.09E-07
ITGBL1	0.708582	1.691907	1.255644	2.15E-13
SLC7A4	3.723111	1.363827	-1.44885	8.11E-23
RPE65	0.441134	0.147738	-1.57818	3.90E-19
SALL4	0.252088	0.53262	1.079178	1.37E-11
RORB-AS1	0.221379	0.444139	1.004497	0.012321
ESM1	1.021666	2.142874	1.068624	5.73E-09
SNORD15B	1.097854	3.137448	1.514905	0.002757
CDCA3	0.452861	0.942392	1.057261	2.35E-21
MYBL2	1.560929	4.56679	1.548775	6.83E-16
GPR88	0.275433	0.772758	1.488317	0.01408
SGK2	0.215306	0.554433	1.364629	6.91E-10
MELK	0.631154	1.542669	1.289364	1.36E-18
COL5A2	3.667335	7.923351	1.111379	4.58E-14
KLHL14	0.481656	0.209202	-1.2031	8.15E-05
ACTA1	52.04519	14.36988	-1.85672	7.44E-05
MUC6	12.33505	3.236517	-1.93025	0.009871
KNL1	0.16245	0.331587	1.029395	2.12E-16

TUBB3	0.139944	0.346464	1.307859	8.06E-14
IAPP	0.177606	0.401523	1.176806	0.000157
CST5	0.149455	0.347047	1.215424	0.000138
FAM111B	1.057159	2.303175	1.123432	1.19E-10
SAA1	19.14188	7.942541	-1.26906	0.000666
SNORA47	0.564573	1.803439	1.675519	0.001772
TPX2	1.781599	4.506391	1.3388	4.20E-22
CEP55	0.551111	1.295354	1.232933	3.56E-19
CENPU	1.678386	3.379932	1.009919	2.73E-18
NCAPG	0.390378	0.960336	1.298666	6.18E-18
SAA2-SAA4	0.666144	0.270022	-1.30276	9.63E-05
MIR4677	0.183258	0.377188	1.04141	3.89E-05
CLNK	0.176112	0.361356	1.036931	0.001266
NKX6-1	0.173812	0.525639	1.596542	3.23E-08
ASPM	0.190474	0.469177	1.300535	2.69E-18
ETV1	4.740989	9.494414	1.001891	0.00909
CA14	0.809713	0.332566	-1.28377	2.54E-16
CDCA5	1.047289	2.25692	1.107695	1.31E-18
FAHD2P1	0.06439	0.627623	3.284997	8.22E-05
SHISA2	1.449423	2.914902	1.007969	5.47E-10
TG	3.016927	0.745082	-2.01761	0.01201
KRTAP5-AS1	0.132926	0.319059	1.263204	1.41E-11
RAD54L	0.302442	0.734407	1.279922	2.46E-20
MND1	0.444181	0.967229	1.122708	4.15E-17
ORM1	190.3702	62.49215	-1.60706	1.05E-06
HJURP	0.805873	1.876674	1.219554	2.74E-14
PROK1	4.144624	1.533433	-1.43448	1.67E-17
OPRK1	1.364819	2.778442	1.025566	0.008086
PTTG1	1.774692	3.767921	1.0862	8.12E-19
EFNA2	0.138207	0.436795	1.660127	5.92E-11
HNRNPA1P57	0.994062	2.730304	1.457653	0.000139
RNA5SP18	0.500745	1.554517	1.634319	0.005603
RNVU1-27	1.244521	2.852275	1.196522	7.05E-14
ORC6	0.332072	0.760381	1.195225	4.19E-24
TNMD	0.278771	0.096009	-1.53784	1.49E-11
LINC00482	0.140188	0.329582	1.23328	1.71E-05
WNT5A	3.449353	7.318071	1.085138	1.98E-06
ASF1B	1.577752	3.343279	1.083393	3.49E-16
SLN	6.626127	2.10115	-1.65699	0.000539
MIR219A1	0.42167	0.975426	1.209919	0.000735
BMP6	2.231271	5.629426	1.335122	3.47E-13
KIF2C	0.717586	1.596347	1.153551	5.31E-23

ASPN	3.845752	8.771866	1.189618	2.22E-19
SPC24	0.879256	1.768212	1.007937	3.33E-14
KRTAP5-1	0.159927	0.398149	1.315894	1.42E-18
PLK1	0.65485	1.554288	1.247017	2.39E-23
ACTC1	8.242348	3.582226	-1.2022	1.09E-09
NMU	0.168529	0.353705	1.069554	1.61E-05
SCARNA12	0.656828	1.615515	1.298406	0.002704
LINC01139	0.421522	0.200424	-1.07255	0.001781
EMX2OS	0.496519	0.089781	-2.46737	0.001092
LDB3	5.080703	2.522899	-1.00995	1.36E-08
NCAPH	0.563164	1.402301	1.316168	6.45E-21
PIMREG	0.502459	1.019327	1.020538	2.89E-15
EFNA3	1.633979	3.440147	1.074081	4.87E-12
SCARNA6	0.558257	1.546154	1.469682	0.01462
PNMA5	0.143017	1.643456	3.522476	0.000333
H2BC17	0.372083	0.784636	1.076397	4.16E-07
SNORA22	0.502403	1.950896	1.957221	0.000141
HMGB1P24	0.424479	0.893484	1.073748	0.007663
CHI3L2	3.402882	1.100922	-1.62805	1.12E-06
DIRAS2	0.228205	0.77814	1.769702	4.73E-10
E2F2	0.205716	0.447416	1.120961	2.58E-14
MYOM1	2.661104	1.28301	-1.05249	0.003712
TPSP2	0.15625	0.420196	1.427207	7.75E-05
PPFIA2	1.011708	2.299822	1.184729	1.03E-06
MYH6	0.323556	0.080973	-1.99851	1.40E-05
DYTN	0.188108	0.537905	1.515791	1.89E-05
AKR1C3	1.0098	2.40764	1.25355	8.55E-09
TACC3	1.505829	3.029582	1.008561	1.22E-19
SYNGR3	0.144817	0.373093	1.365306	4.06E-10
SEMG1	979.5371	41.47477	-4.56179	0.012039
KIF23	0.377162	0.81381	1.109508	1.24E-21
FRMD6-AS2	0.358214	0.175871	-1.02631	2.28E-10
C22orf42	1.376975	0.668841	-1.04177	0.000385
DNAH8	1.705066	3.7181	1.124738	2.03E-05
DPEP1	0.14975	0.906733	2.598117	6.41E-05
COL10A1	1.277545	4.835429	1.92027	6.08E-15
CARMN	0.753241	0.351809	-1.09832	0.002494
SLC5A8	0.589571	0.232681	-1.34131	4.93E-16
HHATL	0.286432	0.110838	-1.36975	2.40E-06
MYOT	2.542854	0.967258	-1.39448	0.005756
MTND4P24	2.077225	0.854378	-1.28171	0.003851
GOLGA7B	0.245781	0.564299	1.199084	1.04E-10
VGF	0.637152	1.409071	1.145035	2.00E-10

ANKRD34B	1.602798	3.418237	1.09266	0.019756
SNORD63	0.233802	0.533543	1.190317	9.27E-05
SFRP4	14.46611	33.65955	1.218338	2.24E-12
COMP	13.02974	29.41427	1.174708	2.36E-10
EOMES	0.218177	0.498108	1.190961	1.44E-06
CAV3	0.283676	0.122572	-1.21062	0.000626
PKMYT1	0.567181	1.272255	1.165508	6.78E-18
TYRP1	0.186995	0.431376	1.205943	0.022079
MCIDAS	0.186575	0.374496	1.005192	0.00735
MIR4666A	0.307204	0.620952	1.015286	7.61E-09
TMEM59L	0.744948	1.535972	1.043941	4.23E-06
CDKN3	0.815693	1.851189	1.182354	1.58E-19
DEPDC1B	0.336012	0.751248	1.160776	1.82E-15
UHRF1	0.426684	0.983495	1.204751	2.70E-13
ETV4	21.00811	1.148106	-4.19362	0.000615
THBS2	3.887035	9.961235	1.357654	1.50E-15
NUSAP1	1.927652	4.624575	1.262476	4.77E-22
TUNAR	0.211586	0.583419	1.463285	0.015263
UGT2B15	0.475475	1.245938	1.38979	3.68E-06
MIR320B2	0.152722	0.31382	1.039034	7.65E-06
COL11A1	0.070939	0.470216	2.728669	1.07E-14
IGSF1	0.433667	0.159999	-1.43853	1.19E-18
CENPI	0.166035	0.365352	1.137796	1.75E-18
JPH4	3.11519	1.506496	-1.04812	9.43E-14
SULT1C3	0.480028	0.207661	-1.20889	0.005985
SUCNR1	0.225442	1.523715	2.756768	2.26E-05
CPHL1P	0.151305	0.343172	1.181469	7.69E-12
SNORA28	0.348586	0.85486	1.294174	1.60E-05
GLP1R	0.145457	0.638929	2.135062	0.011165
NAALADL2-AS2	0.183863	0.751201	2.030568	1.21E-05
SGCG	0.870779	0.39216	-1.15087	2.08E-07
KRT81	0.293944	0.658501	1.163647	0.004862
KIF15	0.343091	0.795053	1.21246	1.39E-21
CKAP2L	0.203548	0.519604	1.352043	6.12E-19
RNA5SP46	0.125956	0.320763	1.348591	4.52E-05
AURKB	0.781991	1.671124	1.095595	1.50E-13
AKR1C1	0.39272	0.802667	1.031301	0.022447
EME1	0.222617	0.461167	1.050725	5.60E-23
MTFR2	0.161506	0.338403	1.067157	9.44E-16
SH2D1A	0.87005	1.831941	1.074203	0.020758
NUF2	0.323876	0.83297	1.362823	5.21E-23
ARHGDIG	1.092831	2.349199	1.104099	1.34E-11

RRM2	1.240677	2.943587	1.246447	7.45E-16
SLC18A2	0.803886	0.38462	-1.06356	0.000352
AQP2	3.346954	0.132486	-4.65894	0.000515
FOXM1	1.006718	2.335529	1.21409	5.48E-21
KLHL41	4.983848	1.690752	-1.55959	0.01296
COL1A1	35.44239	82.34219	1.216156	2.53E-17
DLGAP5	0.453574	1.140549	1.330319	3.38E-20
ESPL1	0.190061	0.491662	1.371202	2.54E-23
CST2	12.11295	42.23007	1.801721	4.61E-10
ATP5MC2P4	0.255294	0.122751	-1.05642	0.00167
OIP5	0.462817	0.9548	1.044755	8.88E-20
E2F8	0.147122	0.307661	1.064331	5.18E-14
MT1L	21.25112	9.726231	-1.12759	9.67E-18
MMP11	0.966709	3.386756	1.808751	2.47E-17
BIRC5	1.387638	3.375586	1.282507	1.08E-15
SPAG5	0.960758	2.025614	1.076114	2.87E-24
TROAP	0.472923	1.301732	1.460755	9.82E-26
KIF20A	0.68902	1.76325	1.35562	1.01E-19
C1QL1	0.885483	0.364014	-1.28247	0.000376
TICRR	0.149916	0.342402	1.191543	3.07E-26
BHLHE40-AS1	0.440095	0.91785	1.060443	3.62E-06
ALB	1.744042	4.228231	1.27762	0.009684
NEK2	0.469256	1.358835	1.533924	3.74E-21
PBK	0.64827	1.619386	1.32078	5.23E-15
HMMR	0.720212	1.533031	1.089894	9.54E-16
DTL	0.429557	0.898525	1.064711	8.95E-13
SPOCK2	3.717194	12.67562	1.769771	8.94E-07
ASIC2	0.163258	0.371228	1.185156	0.000747
GTSE1	0.430398	1.000045	1.216322	4.80E-22
UBE2C	2.272643	7.083286	1.640047	4.55E-22
INHBA	1.068562	2.594225	1.279633	1.26E-14
COL11A2	0.17446	0.371901	1.092025	4.20E-14
SKA3	0.520879	1.129221	1.116309	4.71E-15
CPS1	0.505266	1.416432	1.487145	0.010254
STAC2	2.48413	1.09362	-1.18363	0.001117
LY6G6C	0.139332	0.83963	2.591227	0.00034
CPNE6	0.639982	0.271542	-1.23686	7.37E-17
MYOZ1	3.343483	1.458601	-1.19677	0.021197
NOX4	0.267816	0.582235	1.12036	1.23E-13
PPP1R21-DT	0.423392	0.857518	1.018174	0.000402
FAP	0.487651	0.992337	1.024981	2.01E-12
KCNMB2-	0.181304	0.378393	1.061478	0.014235

AS1				
CDK1	1.451218	3.342402	1.203621	8.28E-18
LINC01612	0.183022	1.180436	2.68923	6.12E-06
SLCO1A2	0.436803	0.973654	1.156428	0.000513
ACRP1	0.238456	0.857206	1.845916	0.000291
VIP	0.295751	1.048726	1.826182	0.001447
PAH	0.451535	0.196313	-1.20168	2.72E-06
GABRD	0.376831	0.786856	1.062183	2.42E-13
RPS26P47	2.639331	0.417485	-2.66038	4.93E-07
SHCBP1	0.279155	0.687733	1.300781	3.85E-20
CTHRC1	2.141064	6.17444	1.52798	9.42E-17
NETO1	0.083695	0.834337	3.317411	5.56E-07
HASPIN	0.146569	0.302762	1.046603	1.89E-15
CELSR3	0.353833	0.960772	1.441125	2.88E-10
USP32P3	0.077335	0.383605	2.310419	2.24E-16
CSAG1	0.113793	0.483856	2.08817	9.47E-06
SAA2	2.386543	0.904954	-1.39901	0.00013
MYT1	0.116311	0.48358	2.055766	2.40E-06
KIF18B	0.225514	0.725717	1.686191	2.07E-23
PGM5-AS1	14.74108	6.706618	-1.13618	2.44E-17
CENPF	0.605913	1.54031	1.346038	1.88E-21
C11orf53	0.178466	0.400695	1.166857	0.000237
KIF4A	0.651416	1.734443	1.412822	6.55E-20
C5orf46	0.250257	0.595474	1.250627	7.74E-05
FNDC1	0.1925	0.546181	1.504519	3.21E-08
STMN2	0.60425	1.85061	1.614783	3.16E-10
UTS2B	0.207355	0.711044	1.777839	5.02E-12
KIFC1	1.330168	2.942513	1.14544	4.42E-22
MIR1-1HG	0.333044	0.114531	-1.53998	0.008784
LIX1	0.502135	0.21942	-1.19438	5.68E-16
KCNK17	0.139788	0.303644	1.11914	3.17E-08
CASQ1	3.009451	1.050889	-1.51789	8.76E-06
SCARNA8	0.291579	0.67302	1.206761	0.000129
CENPE	0.298879	0.61402	1.038724	8.12E-21
DSCAM-AS1	2.188355	4.78321	1.128132	0.009925
EXO1	0.240148	0.619962	1.368255	1.97E-17
RNVU1-29	0.336874	0.841321	1.320446	2.91E-05
MIR5691	0.176148	0.646732	1.876381	7.05E-06
IQGAP3	0.411035	1.159588	1.49628	9.15E-23
PCLAF	0.980376	1.966337	1.004104	7.57E-15
GRIN3A	2.236583	4.992491	1.158463	0.000171
DNAAF3-AS1	0.315344	0.12131	-1.37823	7.64E-05
CD38	11.08372	4.211484	-1.39604	1.29E-17

PNMT	1.08627	0.349377	-1.63652	0.00088
MIR3153	0.282423	0.583111	1.045914	0.000587
RNVU1-15	0.524984	1.471315	1.486761	1.21E-06
VWA5B1	0.150386	0.523748	1.800199	0.026377
ANKRD30A	0.163082	0.417785	1.357166	0.007759
FBXL16	1.346432	3.523681	1.387942	4.38E-11
KLK14	2.86048	10.84427	1.922603	0.006543
ADAMTS9-AS1	0.782706	0.38625	-1.01894	1.09E-13
NKX3-2	0.147221	0.667454	2.180686	2.10E-05
MMP3	0.161032	0.413976	1.362203	0.000381
IP6K3	1.128758	0.529893	-1.09096	2.64E-12
MKI67	1.0716	2.20666	1.042098	1.39E-15
TOP2A	1.816205	4.605807	1.342527	3.70E-18
CKM	21.83134	6.610367	-1.7236	2.04E-11
KRT75	0.063552	0.426769	2.747441	1.64E-06
CBX2	0.644991	1.633776	1.340858	4.06E-21
SYNDIG1L	0.158735	0.337682	1.089047	2.72E-06
SLC13A2	0.40947	0.108557	-1.91531	0.025253
PEBP4	31.19473	14.84206	-1.07161	1.69E-17
ORM2	34.29301	14.69475	-1.22261	1.86E-08
AQP5	0.488308	0.215835	-1.17786	6.45E-14
LINC02718	0.207967	0.422724	1.023362	1.15E-07
SERPINA5	3.756557	0.504583	-2.89625	3.76E-06
NRIP3	0.467504	1.128462	1.271308	1.01E-07
AMH	0.462543	1.456614	1.654958	1.16E-12
B3GNT6	3.276735	6.967217	1.088323	0.003855
MT1B	0.350214	0.160073	-1.12951	1.79E-11
LRRC31	1.053243	2.464012	1.226171	0.000288
RNU6-1010P	0.691392	0.305134	-1.18006	0.000189
IGFL1	0.168114	0.44448	1.402681	0.000195
SGO1	0.162242	0.40817	1.331024	3.62E-19
TNNI1	4.945368	1.636717	-1.59527	0.019284
CCNA2	1.115731	2.47662	1.150384	6.79E-20
OMP	0.166004	0.426696	1.36199	1.61E-05
SCHLAP1	4.172546	12.24687	1.553413	0.000387
ANLN	0.56557	1.305673	1.207017	2.53E-16
LINC01614	0.123279	0.357096	1.534385	5.05E-06
CDCA2	0.194438	0.440155	1.178702	8.77E-11

Supplementary Tabel 2 283 DEGs when comparing Gleason ≤7 samples to Gleason >7 samples

gene	Gleason≤7 Samlpe Mean	Gleason>7 Samlpe Mean	logFC	pValue
SPC25	0.531734	1.162491	1.128443	3.58E-15
PPP1R1A	1.079668	0.535007	-1.01296	3.22E-11
ODF3L2	0.165456	0.333184	1.009872	1.06E-05
INSL5	0.234476	1.398308	2.576172	1.27E-05
CDC25C	0.278669	0.649581	1.220955	2.58E-18
CDC20	1.799942	4.530641	1.331765	1.67E-19
MUCL1	0.506915	4.8361	3.254029	0.001335
ITLN1	0.328921	0.147271	-1.15927	3.66E-06
BUB1	0.453121	1.125699	1.312853	1.17E-21
TTK	0.66106	1.368288	1.049519	5.69E-13
FRG2HP	1.679397	0.780353	-1.10575	0.011227
PRAME	0.242906	0.659258	1.440441	0.011302
MCM10	0.144134	0.36719	1.349116	3.07E-15
CENPA	0.408133	1.186134	1.539155	4.74E-22
RUNDC3A	0.217651	0.505356	1.215286	1.85E-05
SYT4	0.601135	2.551671	2.085682	0.002585
SSTR1	3.054463	8.437271	1.465858	2.09E-07
ITGBL1	0.708582	1.691907	1.255644	2.15E-13
SLC7A4	3.723111	1.363827	-1.44885	8.11E-23
RPE65	0.441134	0.147738	-1.57818	3.90E-19
SALL4	0.252088	0.53262	1.079178	1.37E-11
RORB-AS1	0.221379	0.444139	1.004497	0.012321
ESM1	1.021666	2.142874	1.068624	5.73E-09
SNORD15B	1.097854	3.137448	1.514905	0.002757
CDCA3	0.452861	0.942392	1.057261	2.35E-21
MYBL2	1.560929	4.56679	1.548775	6.83E-16
GPR88	0.275433	0.772758	1.488317	0.01408
SGK2	0.215306	0.554433	1.364629	6.91E-10
MELK	0.631154	1.542669	1.289364	1.36E-18
COL5A2	3.667335	7.923351	1.111379	4.58E-14
KLHL14	0.481656	0.209202	-1.2031	8.15E-05
ACTA1	52.04519	14.36988	-1.85672	7.44E-05
MUC6	12.33505	3.236517	-1.93025	0.009871

KNL1	0.16245	0.331587	1.029395	2.12E-16
TUBB3	0.139944	0.346464	1.307859	8.06E-14
IAPP	0.177606	0.401523	1.176806	0.000157
CST5	0.149455	0.347047	1.215424	0.000138
FAM111B	1.057159	2.303175	1.123432	1.19E-10
SAA1	19.14188	7.942541	-1.26906	0.000666
SNORA47	0.564573	1.803439	1.675519	0.001772
TPX2	1.781599	4.506391	1.3388	4.20E-22
CEP55	0.551111	1.295354	1.232933	3.56E-19
CENPU	1.678386	3.379932	1.009919	2.73E-18
NCAPG	0.390378	0.960336	1.298666	6.18E-18
SAA2-SAA4	0.666144	0.270022	-1.30276	9.63E-05
MIR4677	0.183258	0.377188	1.04141	3.89E-05
CLNK	0.176112	0.361356	1.036931	0.001266
NKX6-1	0.173812	0.525639	1.596542	3.23E-08
ASPM	0.190474	0.469177	1.300535	2.69E-18
ETV1	4.740989	9.494414	1.001891	0.00909
CA14	0.809713	0.332566	-1.28377	2.54E-16
CDCA5	1.047289	2.25692	1.107695	1.31E-18
FAHD2P1	0.06439	0.627623	3.284997	8.22E-05
SHISA2	1.449423	2.914902	1.007969	5.47E-10
TG	3.016927	0.745082	-2.01761	0.01201
KRTAP5-AS1	0.132926	0.319059	1.263204	1.41E-11
RAD54L	0.302442	0.734407	1.279922	2.46E-20
MND1	0.444181	0.967229	1.122708	4.15E-17
ORM1	190.3702	62.49215	-1.60706	1.05E-06
HJURP	0.805873	1.876674	1.219554	2.74E-14
PROK1	4.144624	1.533433	-1.43448	1.67E-17
OPRK1	1.364819	2.778442	1.025566	0.008086
PTTG1	1.774692	3.767921	1.0862	8.12E-19
EFNA2	0.138207	0.436795	1.660127	5.92E-11
HNRNPA1P57	0.994062	2.730304	1.457653	0.000139
RNA5SP18	0.500745	1.554517	1.634319	0.005603
RNVU1-27	1.244521	2.852275	1.196522	7.05E-14
ORC6	0.332072	0.760381	1.195225	4.19E-24
TNMD	0.278771	0.096009	-1.53784	1.49E-11
LINC00482	0.140188	0.329582	1.23328	1.71E-05
WNT5A	3.449353	7.318071	1.085138	1.98E-06
ASF1B	1.577752	3.343279	1.083393	3.49E-16
SLN	6.626127	2.10115	-1.65699	0.000539
MIR219A1	0.42167	0.975426	1.209919	0.000735
BMP6	2.231271	5.629426	1.335122	3.47E-13
KIF2C	0.717586	1.596347	1.153551	5.31E-23

ASPN	3.845752	8.771866	1.189618	2.22E-19
SPC24	0.879256	1.768212	1.007937	3.33E-14
KRTAP5-1	0.159927	0.398149	1.315894	1.42E-18
PLK1	0.65485	1.554288	1.247017	2.39E-23
ACTC1	8.242348	3.582226	-1.2022	1.09E-09
NMU	0.168529	0.353705	1.069554	1.61E-05
SCARNA12	0.656828	1.615515	1.298406	0.002704
LINC01139	0.421522	0.200424	-1.07255	0.001781
EMX2OS	0.496519	0.089781	-2.46737	0.001092
LDB3	5.080703	2.522899	-1.00995	1.36E-08
NCAPH	0.563164	1.402301	1.316168	6.45E-21
PIMREG	0.502459	1.019327	1.020538	2.89E-15
EFNA3	1.633979	3.440147	1.074081	4.87E-12
SCARNA6	0.558257	1.546154	1.469682	0.01462
PNMA5	0.143017	1.643456	3.522476	0.000333
H2BC17	0.372083	0.784636	1.076397	4.16E-07
SNORA22	0.502403	1.950896	1.957221	0.000141
HMGB1P24	0.424479	0.893484	1.073748	0.007663
CHI3L2	3.402882	1.100922	-1.62805	1.12E-06
DIRAS2	0.228205	0.77814	1.769702	4.73E-10
E2F2	0.205716	0.447416	1.120961	2.58E-14
MYOM1	2.661104	1.28301	-1.05249	0.003712
TPSP2	0.15625	0.420196	1.427207	7.75E-05
PPFIA2	1.011708	2.299822	1.184729	1.03E-06
MYH6	0.323556	0.080973	-1.99851	1.40E-05
DYTN	0.188108	0.537905	1.515791	1.89E-05
AKR1C3	1.0098	2.40764	1.25355	8.55E-09
TACC3	1.505829	3.029582	1.008561	1.22E-19
SYNGR3	0.144817	0.373093	1.365306	4.06E-10
SEMG1	979.5371	41.47477	-4.56179	0.012039
KIF23	0.377162	0.81381	1.109508	1.24E-21
FRMD6-AS2	0.358214	0.175871	-1.02631	2.28E-10
C22orf42	1.376975	0.668841	-1.04177	0.000385
DNAH8	1.705066	3.7181	1.124738	2.03E-05
DPEP1	0.14975	0.906733	2.598117	6.41E-05
COL10A1	1.277545	4.835429	1.92027	6.08E-15
CARMN	0.753241	0.351809	-1.09832	0.002494
SLC5A8	0.589571	0.232681	-1.34131	4.93E-16
HHATL	0.286432	0.110838	-1.36975	2.40E-06
MYOT	2.542854	0.967258	-1.39448	0.005756
MTND4P24	2.077225	0.854378	-1.28171	0.003851
GOLGA7B	0.245781	0.564299	1.199084	1.04E-10
VGF	0.637152	1.409071	1.145035	2.00E-10

ANKRD34B	1.602798	3.418237	1.09266	0.019756
SNORD63	0.233802	0.533543	1.190317	9.27E-05
SFRP4	14.46611	33.65955	1.218338	2.24E-12
COMP	13.02974	29.41427	1.174708	2.36E-10
EOMES	0.218177	0.498108	1.190961	1.44E-06
CAV3	0.283676	0.122572	-1.21062	0.000626
PKMYT1	0.567181	1.272255	1.165508	6.78E-18
TYRP1	0.186995	0.431376	1.205943	0.022079
MCIDAS	0.186575	0.374496	1.005192	0.00735
MIR4666A	0.307204	0.620952	1.015286	7.61E-09
TMEM59L	0.744948	1.535972	1.043941	4.23E-06
CDKN3	0.815693	1.851189	1.182354	1.58E-19
DEPDC1B	0.336012	0.751248	1.160776	1.82E-15
UHRF1	0.426684	0.983495	1.204751	2.70E-13
ETV4	21.00811	1.148106	-4.19362	0.000615
THBS2	3.887035	9.961235	1.357654	1.50E-15
NUSAP1	1.927652	4.624575	1.262476	4.77E-22
TUNAR	0.211586	0.583419	1.463285	0.015263
UGT2B15	0.475475	1.245938	1.38979	3.68E-06
MIR320B2	0.152722	0.31382	1.039034	7.65E-06
COL11A1	0.070939	0.470216	2.728669	1.07E-14
IGSF1	0.433667	0.159999	-1.43853	1.19E-18
CENPI	0.166035	0.365352	1.137796	1.75E-18
JPH4	3.11519	1.506496	-1.04812	9.43E-14
SULT1C3	0.480028	0.207661	-1.20889	0.005985
SUCNR1	0.225442	1.523715	2.756768	2.26E-05
CPHL1P	0.151305	0.343172	1.181469	7.69E-12
SNORA28	0.348586	0.85486	1.294174	1.60E-05
GLP1R	0.145457	0.638929	2.135062	0.011165
NAALADL2-AS2	0.183863	0.751201	2.030568	1.21E-05
SGCG	0.870779	0.39216	-1.15087	2.08E-07
KRT81	0.293944	0.658501	1.163647	0.004862
KIF15	0.343091	0.795053	1.21246	1.39E-21
CKAP2L	0.203548	0.519604	1.352043	6.12E-19
RNA5SP46	0.125956	0.320763	1.348591	4.52E-05
AURKB	0.781991	1.671124	1.095595	1.50E-13
AKR1C1	0.39272	0.802667	1.031301	0.022447
EME1	0.222617	0.461167	1.050725	5.60E-23
MTFR2	0.161506	0.338403	1.067157	9.44E-16
SH2D1A	0.87005	1.831941	1.074203	0.020758
NUF2	0.323876	0.83297	1.362823	5.21E-23
ARHGDIG	1.092831	2.349199	1.104099	1.34E-11

RRM2	1.240677	2.943587	1.246447	7.45E-16
SLC18A2	0.803886	0.38462	-1.06356	0.000352
AQP2	3.346954	0.132486	-4.65894	0.000515
FOXM1	1.006718	2.335529	1.21409	5.48E-21
KLHL41	4.983848	1.690752	-1.55959	0.01296
COL1A1	35.44239	82.34219	1.216156	2.53E-17
DLGAP5	0.453574	1.140549	1.330319	3.38E-20
ESPL1	0.190061	0.491662	1.371202	2.54E-23
CST2	12.11295	42.23007	1.801721	4.61E-10
ATP5MC2P4	0.255294	0.122751	-1.05642	0.00167
OIP5	0.462817	0.9548	1.044755	8.88E-20
E2F8	0.147122	0.307661	1.064331	5.18E-14
MT1L	21.25112	9.726231	-1.12759	9.67E-18
MMP11	0.966709	3.386756	1.808751	2.47E-17
BIRC5	1.387638	3.375586	1.282507	1.08E-15
SPAG5	0.960758	2.025614	1.076114	2.87E-24
TROAP	0.472923	1.301732	1.460755	9.82E-26
KIF20A	0.68902	1.76325	1.35562	1.01E-19
C1QL1	0.885483	0.364014	-1.28247	0.000376
TICRR	0.149916	0.342402	1.191543	3.07E-26
BHLHE40-AS1	0.440095	0.91785	1.060443	3.62E-06
ALB	1.744042	4.228231	1.27762	0.009684
NEK2	0.469256	1.358835	1.533924	3.74E-21
PBK	0.64827	1.619386	1.32078	5.23E-15
HMMR	0.720212	1.533031	1.089894	9.54E-16
DTL	0.429557	0.898525	1.064711	8.95E-13
SPOCK2	3.717194	12.67562	1.769771	8.94E-07
ASIC2	0.163258	0.371228	1.185156	0.000747
GTSE1	0.430398	1.000045	1.216322	4.80E-22
UBE2C	2.272643	7.083286	1.640047	4.55E-22
INHBA	1.068562	2.594225	1.279633	1.26E-14
COL11A2	0.17446	0.371901	1.092025	4.20E-14
SKA3	0.520879	1.129221	1.116309	4.71E-15
CPS1	0.505266	1.416432	1.487145	0.010254
STAC2	2.48413	1.09362	-1.18363	0.001117
LY6G6C	0.139332	0.83963	2.591227	0.00034
CPNE6	0.639982	0.271542	-1.23686	7.37E-17
MYOZ1	3.343483	1.458601	-1.19677	0.021197
NOX4	0.267816	0.582235	1.12036	1.23E-13
PPP1R21-DT	0.423392	0.857518	1.018174	0.000402
FAP	0.487651	0.992337	1.024981	2.01E-12
KCNMB2-	0.181304	0.378393	1.061478	0.014235

AS1				
CDK1	1.451218	3.342402	1.203621	8.28E-18
LINC01612	0.183022	1.180436	2.68923	6.12E-06
SLCO1A2	0.436803	0.973654	1.156428	0.000513
ACRP1	0.238456	0.857206	1.845916	0.000291
VIP	0.295751	1.048726	1.826182	0.001447
PAH	0.451535	0.196313	-1.20168	2.72E-06
GABRD	0.376831	0.786856	1.062183	2.42E-13
RPS26P47	2.639331	0.417485	-2.66038	4.93E-07
SHCBP1	0.279155	0.687733	1.300781	3.85E-20
CTHRC1	2.141064	6.17444	1.52798	9.42E-17
NETO1	0.083695	0.834337	3.317411	5.56E-07
HASPIN	0.146569	0.302762	1.046603	1.89E-15
CELSR3	0.353833	0.960772	1.441125	2.88E-10
USP32P3	0.077335	0.383605	2.310419	2.24E-16
CSAG1	0.113793	0.483856	2.08817	9.47E-06
SAA2	2.386543	0.904954	-1.39901	0.00013
MYT1	0.116311	0.48358	2.055766	2.40E-06
KIF18B	0.225514	0.725717	1.686191	2.07E-23
PGM5-AS1	14.74108	6.706618	-1.13618	2.44E-17
CENPF	0.605913	1.54031	1.346038	1.88E-21
C11orf53	0.178466	0.400695	1.166857	0.000237
KIF4A	0.651416	1.734443	1.412822	6.55E-20
C5orf46	0.250257	0.595474	1.250627	7.74E-05
FNDC1	0.1925	0.546181	1.504519	3.21E-08
STMN2	0.60425	1.85061	1.614783	3.16E-10
UTS2B	0.207355	0.711044	1.777839	5.02E-12
KIFC1	1.330168	2.942513	1.14544	4.42E-22
MIR1-1HG	0.333044	0.114531	-1.53998	0.008784
LIX1	0.502135	0.21942	-1.19438	5.68E-16
KCNK17	0.139788	0.303644	1.11914	3.17E-08
CASQ1	3.009451	1.050889	-1.51789	8.76E-06
SCARNA8	0.291579	0.67302	1.206761	0.000129
CENPE	0.298879	0.61402	1.038724	8.12E-21
DSCAM-AS1	2.188355	4.78321	1.128132	0.009925
EXO1	0.240148	0.619962	1.368255	1.97E-17
RNVU1-29	0.336874	0.841321	1.320446	2.91E-05
MIR5691	0.176148	0.646732	1.876381	7.05E-06
IQGAP3	0.411035	1.159588	1.49628	9.15E-23
PCLAF	0.980376	1.966337	1.004104	7.57E-15
GRIN3A	2.236583	4.992491	1.158463	0.000171
DNAAF3-AS1	0.315344	0.12131	-1.37823	7.64E-05
CD38	11.08372	4.211484	-1.39604	1.29E-17

PNMT	1.08627	0.349377	-1.63652	0.00088
MIR3153	0.282423	0.583111	1.045914	0.000587
RNVU1-15	0.524984	1.471315	1.486761	1.21E-06
VWA5B1	0.150386	0.523748	1.800199	0.026377
ANKRD30A	0.163082	0.417785	1.357166	0.007759
FBXL16	1.346432	3.523681	1.387942	4.38E-11
KLK14	2.86048	10.84427	1.922603	0.006543
ADAMTS9-AS1	0.782706	0.38625	-1.01894	1.09E-13
NKX3-2	0.147221	0.667454	2.180686	2.10E-05
MMP3	0.161032	0.413976	1.362203	0.000381
IP6K3	1.128758	0.529893	-1.09096	2.64E-12
MKI67	1.0716	2.20666	1.042098	1.39E-15
TOP2A	1.816205	4.605807	1.342527	3.70E-18
CKM	21.83134	6.610367	-1.7236	2.04E-11
KRT75	0.063552	0.426769	2.747441	1.64E-06
CBX2	0.644991	1.633776	1.340858	4.06E-21
SYNDIG1L	0.158735	0.337682	1.089047	2.72E-06
SLC13A2	0.40947	0.108557	-1.91531	0.025253
PEBP4	31.19473	14.84206	-1.07161	1.69E-17
ORM2	34.29301	14.69475	-1.22261	1.86E-08
AQP5	0.488308	0.215835	-1.17786	6.45E-14
LINC02718	0.207967	0.422724	1.023362	1.15E-07
SERPINA5	3.756557	0.504583	-2.89625	3.76E-06
NRIP3	0.467504	1.128462	1.271308	1.01E-07
AMH	0.462543	1.456614	1.654958	1.16E-12
B3GNT6	3.276735	6.967217	1.088323	0.003855
MT1B	0.350214	0.160073	-1.12951	1.79E-11
LRRC31	1.053243	2.464012	1.226171	0.000288
RNU6-1010P	0.691392	0.305134	-1.18006	0.000189
IGFL1	0.168114	0.44448	1.402681	0.000195
SGO1	0.162242	0.40817	1.331024	3.62E-19
TNNI1	4.945368	1.636717	-1.59527	0.019284
CCNA2	1.115731	2.47662	1.150384	6.79E-20
OMP	0.166004	0.426696	1.36199	1.61E-05
SCHLAP1	4.172546	12.24687	1.553413	0.000387
ANLN	0.56557	1.305673	1.207017	2.53E-16
LINC01614	0.123279	0.357096	1.534385	5.05E-06
CDCA2	0.194438	0.440155	1.178702	8.77E-11

Supplementary Tabel 3 There are 60 overlapping DEGs in both the normal vs. tumor and Gleason ≤7 vs. >7 comparisons.

gene	Gleason score>7 vs <=7		Tumor vs Normal	
	logFC	fdr	logFC	fdr
ITLN1	-1.159266	2.2664E-05	-2.4442951	1.2745E-05
PRAME	1.4404414	0.02309352	2.27501186	9.1138E-07
SSTR1	1.4658576	1.8512E-06	2.04321543	1.5977E-06
RPE65	-1.578176	9.8309E-17	-2.14967	2.703E-17
SNORD15B	1.5149055	0.00694174	4.03381318	5.5732E-06
KLHL14	-1.203104	0.00033322	-3.8774182	1.2259E-14
MUC6	-1.930249	0.02057721	-4.5650133	0.01144087
IAPP	1.1768064	0.00058256	3.25886406	2.0322E-05
NKX6-1	1.5965417	3.6382E-07	3.59232485	4.5002E-09
CA14	-1.283768	2.8794E-14	-2.4707509	9.4127E-20
TG	-2.017608	0.0243234	-2.6233665	4.9318E-08
HJURP	1.2195539	1.7715E-12	2.03387295	2.2728E-18
EFNA2	1.6601267	1.5261E-09	3.11233763	3.4947E-11
HNRNPA1P57	1.4576531	0.00052592	4.55786759	8.1401E-07
TNMD	-1.53784	4.476E-10	-3.0457641	2.4495E-11
KRTAP5-1	1.3158944	3.0382E-16	2.16241636	6.2438E-12
ACTC1	-1.202199	1.9276E-08	-2.3122584	3.6703E-16
SCARNA12	1.2984059	0.00683026	3.13701336	4.6502E-07
LINC01139	-1.072548	0.00475737	-2.9534594	6.2164E-11
EMX2OS	-2.467365	0.00310755	-5.5308027	9.4009E-14
SCARNA6	1.4696819	0.02871648	2.75765081	7.1338E-05
SNORA22	1.9572211	0.00053385	4.23674903	6.1296E-05
HMGB1P24	1.0737482	0.01662152	2.25182801	1.2473E-10
MYH6	-1.998508	7.2972E-05	-2.5742091	3.978E-15
DYTN	1.5157906	9.459E-05	3.26232783	6.5878E-08
SEMG1	-4.561794	0.02437191	-9.2159651	5.6451E-10
DNAH8	1.1247381	0.00010025	2.44849175	1.9873E-08
COL10A1	1.92027	4.5078E-13	2.40124533	6.144E-15
VGF	1.1450353	4.4031E-09	2.57021825	7.874E-13
SNORD63	1.1903167	0.00037259	2.20464389	0.00010276
COMP	1.1747079	5.095E-09	2.08768684	2.2807E-11

MIR4666A	1.015286	1.0382E-07	2.20605832	4.5437E-08
IGSF1	-1.43853	2.6533E-16	-2.2302741	2.0038E-18
GLP1R	2.1350616	0.02285245	2.70548361	0.03287911
NAALADL2-AS2	2.0305679	6.4089E-05	4.26550675	1.8387E-06
ARHGDIG	1.1040993	4.0757E-10	2.77161479	1.0578E-16
SLC18A2	-1.063556	0.00117458	-2.6727	3.5828E-08
AQP2	-4.658937	0.00163467	-8.7999295	4.8385E-14
CST2	1.8017208	9.0632E-09	3.51808658	4.2395E-12
ALB	1.2776198	0.02024213	4.34723034	4.8079E-08
STAC2	-1.18363	0.00317089	-4.0133308	3.3179E-08
CPNE6	-1.236858	9.3742E-15	-2.066327	7.4902E-17
KCNMB2-AS1	1.061478	0.02805213	2.76407089	0.00686466
LINC01612	2.6892304	3.5486E-05	2.35062341	0.03613858
ACRP1	1.8459158	0.00099784	3.23486054	0.00229186
NETO1	3.317411	4.3625E-06	3.75121337	0.00035959
SCARNA8	1.2067612	0.00049465	3.38265267	0.00146813
DSCAM-AS1	1.1281323	0.02066491	2.3908119	0.04518297
RNVU1-29	1.3204459	0.00013699	3.41490818	0.00022472
GRIN3A	1.1584631	0.00062817	2.7259339	0.01203635
PNMT	-1.636524	0.0025854	-4.3530842	2.592E-16
RNVU1-15	1.4867611	8.6501E-06	2.47239208	0.00012886
ANKRD30A	1.3571659	0.01678797	6.27333913	0.00204128
KLK14	1.9226034	0.01449213	2.17139532	0.00050956
SLC13A2	-1.915305	0.04555563	-5.940116	0.00257462
AQP5	-1.177862	3.7891E-12	-2.1667529	3.1857E-17
SERPINA5	-2.896247	2.3248E-05	-6.3538445	9.1005E-17
AMH	1.6549583	4.6558E-11	3.25806469	2.6497E-12
B3GNT6	1.0883234	0.00926165	4.14816367	2.6963E-06
SCHLAP1	1.5534134	0.00127546	2.12632604	0.00165301

Supplementary Tabel 4 Fourteen genes showed statistically significant results in both Kaplan-Meier survival analysis (p-value) and hazard ratio calculations

gene	KM	HR	HR.95L	HR.95H	coxPvalue
RPE65	0.001171	0.224071	0.090527	0.554618	0.001218
IGSF1	0.021462	0.338157	0.143657	0.795991	0.013052
CA14	0.002243	0.339192	0.176181	0.65303	0.001217
SLC18A2	0.044213	0.483518	0.280167	0.834464	0.009056
CPNE6	0.008251	0.545522	0.32055	0.928385	0.025491
COMP	0.003095	1.007073	1.003281	1.01088	0.000251
SSTR1	0.013868	1.013404	1.003443	1.023463	0.00824
LINC01612	0.035492	1.058526	1.01737	1.101346	0.004938
NAALADL2-AS2	0.014973	1.08805	1.041221	1.136986	0.00017
VGF	0.000324	1.116972	1.067105	1.16917	2.06E-06
AMH	3.96E-07	1.129623	1.078766	1.182877	2.15E-07
ARHGDIG	0.007701	1.135306	1.075493	1.198446	4.32E-06
HJURP	6.51E-07	1.227047	1.129005	1.333603	1.47E-06
KRTAP5-1	6.39E-05	2.781904	1.966714	3.934985	7.35E-09

Supplementary Tabel 5 The coefficient of six genes

id	coefficient
SSTR1	0.010264
CA14	-0.57497
HJURP	0.110079
KRTAP5-1	0.491603
VGF	0.071275
COMP	0.005309

Supplementary Tabel 6 The 29 immune signatures represented by 29 different gene libraries

Immune signatures	Gene names
aDCs	CD83 LAMP3 CCL1
APC co inhibition	C10orf54 CD274 LGALS9 PDCD1LG2 PVRL3
APC co stimulation	CD40 CD58 CD70 ICOSLG SLAMF1 TNFSF14 TNFSF15 TNFSF18 TNFSF4 TNFSF8 TNFSF9
B cells	BACH2 BANK1 BLK BTLA CD79A CD79B FCRL1 FCRL3 HVCN1 RALGPS2
CCR	CCL16 TPO TGFBR2 CXCL2 CCL14 TGFBR3 IL11RA CCL11 IL4I1 IL33 CXCL12 CXCL10 BMPER BMP8A CXCL11 IL21R TNFRSF9 IL17B ILF2 CX3CR1 CCR8 TNFSF12 CSF3 TNFSF4 BMP3 CX3CL1 BMP5 CXCR2 TNFRSF10D BMP2 CXCL14 CCL28 CXCL3 BMP6 CCL21 CXCL9 CCL23 IL6 TNFRSF18 IL17RD

	IL17D IL27 CCL7 IL1R1 CXCR4 CXCR2P1 TGFB1I1 IFNGR1 IL9R IL1RAPL1 IL11 CSF1 IL20RA IL25 TNFRSF4 IL18 ILF3 CCL20 TNFRSF12A IL6ST CXCL13 IL12B TNFRSF8 IL6R BMPR2 IFNE IL1RAPL2 IL3RA BMP4 CCL24 TNFSF13B CCR4 IL2RA IL32 TNFRSF10C IL22RA1 BMPR1A CXCR5 CXCR3 IFNA8 IL17REL IFNB1 IFNAR1 TNFRSF1B CCL17 IFNL1 IL16 IL1RL1 ILK CCL25 ILDR2 CXCR1 IL36RN IL34 TGFB1 IFNG IL19 ILKAP BMP2K CCR10 ILDR1 EPO CCR7 IL17C IL23A CCR5 IL7 EPOR CCL13 IL2RG IL31RA TNFAIP6 IFNL2 BMP1 IL12RB1 TNFAIP8 IL4R TNFRSF6B TNFAIP8L1 TNFRSF10B IFNL3 CCL5 CXCL6 CXCL1 CCR3 TNFSF11 CSF1R IL21 IL1RAP IL12RB2 CCL1 IL17RA CCR1 IL1RN TNFRSF11B TNFRSF14 IL13 IL2RB BMP8B CCL2 IL24 IL18RAP TGFBI TNFSF1 TNFRSF11A CXCL5 IL5RA TNFSF9 IL1RL2 TNFRSF13C IL36G IL15RA TNFRSF21 CXCL8 IL22RA2 TNFAIP8L2 IL18R1 IFNLR1 CXCR6 CCL3L3 TNFRSF1A IL17RE IFNGR2 IL17RC TNFAIP8L3 ILVBL TGFBRAP1 CCL4L1 CSF2RA CCRN4L CCL26 TNFAIP1 CCRL2 IFNA10 TNFRSF17 IFNA13 IL20 IL18BP CCL3L1 TNFSF12-TNFSF13 IL5 IL23R IL26 TNF TGFA CSF2 IL1F10 CXCL17 TNFSF13 IFNA4 IL37 IL12A IL7R IFNA1 IL1A IL4 IL2 CCL22 CSF3R IL10 IFNK TGFB2 IL1R2 IL1B IL17F IL27RA IL15 TNFSF8 IL36B XCL1 CXCL16 TNFRSF19 IL3 CCL3 IFNA2 BMPR1B IFNA21 TNFSF18 CCL8 IL17RB TNFRSF25 IL22 IL10RB IFNAR2 CCL18 IFNA16 CSF2RB IL36A TNFAIP3 IL13RA2 IL13RA1 CCR9 TNFRSF10A IFNA7 IFNW1 XCL2 TNFSF14 CCR2 BMP15 BMP10 CCL15 CCL14 TGFBR1 IFNA5 BMP7 IFNA14 IL20RB IL10RA IFNA17 CCR6 TGFB3 CCL15 CCL4 CCL27 TNFRSF13B TNFAIP2 IL31 IL17A TNFSF15 CCL19 IFNA6 IL9
CD8+ T cells	CD8A
Check-point	IDO1 LAG3 CTLA4 TNFRSF9 ICOS CD80 PDCD1LG2 TIGIT CD70 TNFSF9 ICOSLG KIR3DL1 CD86 PDCD1 LAIR1 TNFRSF8 TNFSF15 TNFRSF14 IDO2 CD276 CD40 TNFRSF4 TNFSF14 HHLA2 CD244 CD274 HAVCR2 CD27 BTLA LGALS9 TMIGD2 CD28 CD48 TNFRSF2 CD40LG ADORA2A VTCN1 CD160 CD44 TNFSF18 TNFRSF18 BTNL2 C10orf54 CD200R1 TNFSF4 CD200 NRP1
Cytolytic activity	PRF1 GZMA
DCs	CCL17 CCL22 CD209 CCL13
HLA	HLA-E HLA-DPB2 HLA-C HLA-J HLA-DQB1 HLA-DQB2 HLA-DQA2 HLA-DQA1 HLA-A HLA-DMA HLA-DOB HLA-DRB1 HLA-H
iDCs	CD1A CD1E
Inflammation-promoting	CCL5 CD19 CD8B CXCL10 CXCL13 CXCL9 GNLY GZMB IFNG IL12A IL12B IRF1 PRF1 STAT1 TBX21
Macrophages	C11orf45 CD68 CLEC5A CYBB FUCA1 GPNMB HS3ST2 LGMN MMP9 TM4SF19
Mast cells	CMA1 MS4A2 TPSAB1
MHC class I	B2M HLA-A TAP1
Neutrophils	EVI2B HSD17B11 KDM6B MEGF9 MNDA NLRP12 PADI4 SELL TRANK1 VNN3
NK cells	KLRC1 KLRF1
Parainflammation	CXCL10 PLAT CCND1 LGMN PLAUR AIM2 MMP7 ICAM1 MX2 CXCL9 ANXA1 TLR2 PLA2G2D ITGA2 MX1 HMOX1 CD276 TIRAP IL33 PTGES TNFRSF12A SCARB1 CD14 BLNK IFIT3 RETNLB IFIT2 ISG15 OAS2 REL OAS3 CD44 PPARG BST2 OAS1 NOX1 PLA2G2A IFIT1 IFITM3 IL1RN
pDCs	CLEC4C CXCR3 GZMB IL3RA IRF7 IRF8 LILRA4 PHEX PLD4 PTCRA
T cell co-inhibition	BTLA C10orf54 CD160 CD244 CD274 CTLA4 HAVCR2 LAG3 LAIR1 TIGIT
T cell co-stimulation	CD2 CD226 CD27 CD28 CD40LG ICOS SLAMF1 TNFRSF18 TNFRSF25 TNFRSF4 TNFRSF8 TNFRSF9 TNFSF14
T helper cells	CD4

Tfh	PDCD1 CXCL13 CXCR5
Th1 cells	IFNG TBX21 CTLA4 STAT4 CD38 IL12RB2 LTA CSF2
Th2 cells	PMCH LAIR2 SMAD2 CXCR6 GATA3 IL26
TIL	ITM2C CD38 THEMIS2 GLYR1 ICOS F5 TIGIT KLRD1 IRF4 PRKCQ FCRL5 SIRPG LPXN IL2RG CCL5 LCK TRAF3IP3 CD86 MAL LILRB1 DOK2 CD6 PAG1 LAX1 PLEK PIK3CD SLAMF1 XCL1 GPR171 XCL2 TBX21 CD2 CD53 KLHL6 SLAMF6 CD40 SIT1 TNFRSF4 CD79A CD247 LCP2 CD3D CD27 SH2D1A FYB ARHGAP30 ACAP1 CST7 CD3G IL2RB CD3E FCRL3 CORO1A ITK TCL1A CYBB CSF2RB IKZF1 NCF4 DOCK2 CCR2 PTPRC PLAC8 NCKAP1L IL7R CD28 STAT4 CD8A LY9 CD48 HCST PTPRCAP SASH3 ARHGAP25 LAT TRAT1 IL10RA PAX5 CCR7 DOCK11 PARVG SPNS1 CD52 HCLS1 ARHGAP9 GIMAP6 PRKCB MS4A1 GPR18 TBC1D10C GVINP1 P2RY8 EVI2B VAMP5 KLRK1 SELL MPEG1 MS4A6A ARHGAP15 MFNG GZMK SELPLG TARP GIMAP7 FAM65B INPP5D ITGA4 MZB1 GPSM3 STK10 CLEC2D IL16 NLRC3 GIMAP5 GIMAP4 IFFO1 CFH PVRIG CFHR1
Treg	IL12RB2 TMPRSS6 CTSC LAPTM4B TFRC RNF145 NETO2 ADAT2 CHST2 CTLA4 NFE2L3 LIMA1 IL1R2 ICOS HSDL2 HTATIP2 FKBP1A TIGIT CCR8 LTA SLC35F2 IL21R AHCYL1 SOCS2 ETV7 BCL2L1 RRAGB ACSL4 CHRNA6 BATF LAX1 ADPRH TNFRSF4 ANKRD10 CD274 CASP1 LY75 NPTN SSTR3 GRSF1 CSF2RB TMEM184C NDFIP2 ZBTB38 ERI1 TRAF3 NAB1 HS3ST3B1 LAYN JAK1 VDR LEPROT GCNT1 PTPRJ IKZF2 CSF1 ENTPD1 TNFRSF18 METTL7A KSR1 SSH1 CADM1 IL1R1 ACP5 CHST7 THADA CD177 NFAT5 ZNF282 MAGEH1
Type I IFN Reponse	DDX4 IFIT1 IFIT2 IFIT3 IRF7 ISG20 MX1 MX2 RSAD2 TNFSF10
Type II IFN Reponse	GPR146 SELP AHR

Supplementary Tabel 7 RT-qPCR primers sequences and siRNA sequences

primer			
		F	R
	homo-CA14	5'-ACAGAACTACCGAGCCCTTCAG-3'	5'-GCAGAGACAGCCAACCAAGATTC-3'
	homo-HJURP	5'-GCGGCTGATAGAGAAGTACAACCA-3'	5'-CAATCCCTGTGGCGTCTCGTA-3'
	homo-VGF	5'-CTGTCCACCAAACTCCACCTG-3'	5'-TTCTTCTTCCGCTTCCGCTTC-3'
	homo-COMP	5'-ACAGTGATGGCGATGGTATAGG-3'	5'-CACAAGCATCTCCCACAAAGTC-3'
	homo-KRTAP5-1	5'-GTGTGTTGCTGTTCCTGTTCCA-3'	5'-GGCTTGCAGCAGCTGGATTG-3'
	homo-SSTR1	5'-TAATGGTGATGATGGTGGTGATGG-3'	5'-ATAGCCGAGGATGACCGACAG-3'
siRNA		S	AS

	siVGF-1	AGACGAUCGACAGCCUCAUTT	AUGAGGCUGUCGAUCGUCUTT
	siVGF-2	AAGCGGAAGCGGAAGAAGATT	UCUUCUUCCGCUUCCGCUUTT
